# Two-Step Flow Amidation of Natural Phenolic Acids as Antiradical and Antimicrobial Agents

**DOI:** 10.1021/acs.jnatprod.5c00131

**Published:** 2025-03-31

**Authors:** Desirée Pecora, Anna M. Magni, Sara Vicinanza, Francesca Annunziata, Salvatore Princiotto, Silvia Donzella, Gabriele Meroni, Piera A. Martino, Nicoletta Basilico, Silvia Parapini, Paola Conti, Chiara Borsari, Lucia Tamborini

**Affiliations:** † Department of Pharmaceutical Sciences, University of Milan, Via Mangiagalli 25, 20133 Milan, Italy; ‡ Department of Food, Environmental and Nutritional Sciences, University of Milan, Via Celoria 2, 20133 Milan, Italy; § Department of Biomedical, Surgical and Dental Sciences, One Health Unit, University of Milan, Via Pascal 36, 20133 Milan, Italy; ∥ Department of Biomedical Sciences of Health, University of Milan, Via Pascal 36, 20133 Milan, Italy

## Abstract

Natural hydroxycinnamic acid amides (HCAAs) and riparins offer significant health benefits. However, their extraction from natural sources is difficult, and traditional synthetic methods remain wasteful, raising the need for more efficient alternatives. In this work, a two-step chemo-enzymatic flow method for the efficient esterification and amidation of phenolic acids was developed and successfully applied to the synthesis of riparin derivatives and HCAAs. The flow Fischer esterification was optimized using vanillic acid as a model starting material and SiliaBond Tosic Acid (SCX-3) as an immobilized acid catalyst, achieving a quantitative yield in a short residence time. The following amidation step, catalyzed by immobilized *Candida antarctica* lipase B, was optimized in toluene, leading to the desired amides. The synthesized compounds were evaluated for their radical scavenging, antibacterial, and antileishmanial properties. Overall, this work disclosed a novel approach for the efficient synthesis of riparin derivatives and HCAAs with interesting biological properties.

## Graphical abstract

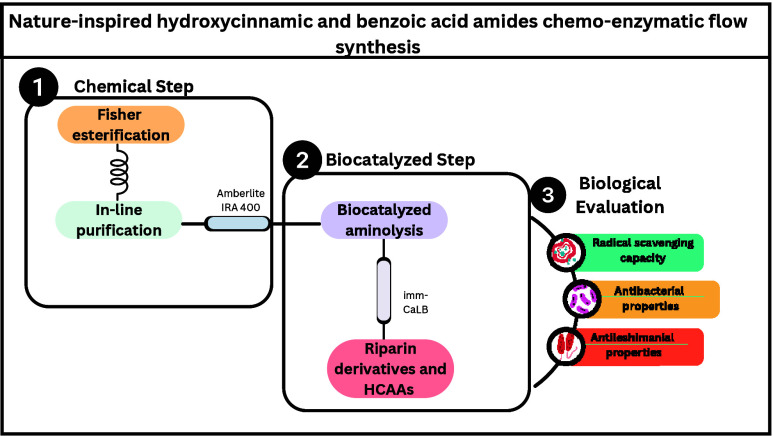

Phenolic acids are aromatic secondary plant metabolites widely spread throughout the plant kingdom.^1^ Depending on their structure, phenolic acids can be divided into two classes: benzoic acid derivatives and cinnamic acid derivatives.^2^ Usually, they could be found as esters, amides, or glycosides, but rarely in free form.^3^ Among amides, natural hydroxycinnamic acid amides (HCAAs) and riparins are alkylamides derived from the conjugation of amines with hydroxycinnamic and benzoic acids, respectively. They have gained increasing attention due to their health benefits,^4–6^ such as antioxidant,^7–9^ anti-inflammatory,^10^ antidiabetic,^11^ antiparasitic,^12–14^ antibacterial,^15^ and anticancer activity.^16,17^ Although they occur in many plants, their extraction from natural sources is challenging due to the complexity of the extraction mixtures, which often require the consumption of substantial amounts of solvents and plant materials. Moreover, the substances of interest are often present in very low amounts, and their availability can be influenced by a variety of factors including seasonal variability, growth stage, climate, soil composition, and the specific part of the plant used for extraction. Therefore, synthesizing pure natural amides and their derivatives is essential for advancing studies on their bioactivity and exploring structure–activity relationships.

The conversion of phenolic acids into the corresponding esters or amides typically involves three or four synthetic steps: (i) protection of phenolic hydroxy groups, (ii) activation of the carboxylic acid group by forming acyl chloride or using coupling reagents such as phosphonium and uronium salts or carbodiimides in the presence of additives such as HOBt and HOAt, (iii) condensation with alcohols or amines, and (iv) deprotection of phenolic hydroxy groups.^18–21^ These protocols use stoichiometric coupling reagents, additives, strong acids, high temperatures, and long reaction times, leading to complex workup procedures that produce high amounts of waste. Although more convenient alternative methods have emerged,^22,23^ most of these techniques often produce phenolic esters or amides in low yield.^24–26^ Several chemical catalysts, such as those based on iron, boron, and aluminum, have been reported for amide bond formation, but they often require high temperatures, complicated post-treatment, or toxic preparation processes.^27–31^ This underscores the need for a more versatile, convenient, and high-yielding method to produce phenolic acid esters and amides.^32,33^ In this context, enzymatic approaches for amide synthesis (e.g., hydrolases, acyltransferases, ATP-dependent enzymes) hold promise for creating shorter, more efficient and sustainable routes to synthesize small-molecule pharmaceutical ingredients.^34–38^ Lipases catalyze transacylation reactions, promoting the aminolysis of esters to obtain amides, and may enable the synthesis of both natural and unnatural derivatives, thus expanding the potential for pharmacologically active compounds.^39^ However, in contrast to the traditional chemical methods for synthesizing HCAAs and riparins, enzyme-catalyzed synthesis has been poorly investigated to date.^36,40^ Importantly, also the esterification of phenolic acids with alcohols can be efficiently achieved through enzymatic catalysis using lipases, as demonstrated in several studies;^41,42^ however, in this study we selected an acid-catalyzed approach to increase the versatility and the possibility to extensively recover and reuse the immobilized catalyst.

In this paper, we report a novel two-step chemo-enzymatic flow method for the esterification and amidation of phenolic acids to obtain natural or nature-inspired esters and amides. The developed protocol was applied to the synthesis of some riparin derivatives (compounds **1**–**4**) and HCAAs (compounds **5**–**10**) that were evaluated for their radical scavenger, antibacterial, and antileishmanial properties.
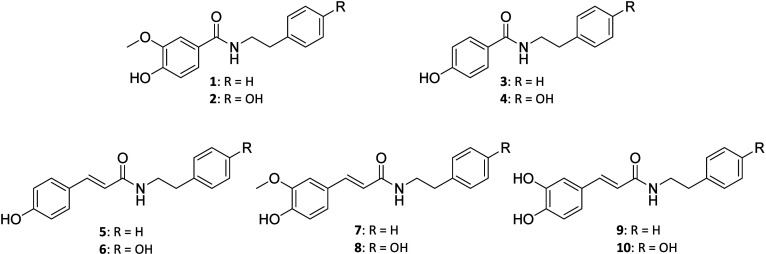


## Results and Discussion

First, the flow Fischer esterification was studied using vanillic acid as the model starting material and Silia*Bond* Tosic Acid (SCX-3) as the immobilized acid catalyst. A solution of vanillic acid (0.2 M) in MeOH was pumped by a syringe pump through a packed-bed column reactor containing the resin SCX-3, and different temperatures (i.e., 65 °C, 70 °C) and residence times (i.e., 90 min, 60 min, 30 min) were investigated. A back-pressure regulator (40 psi) was used to pressurize the system. After collection of the exiting flow and solvent evaporation, the crude product was dissolved in EtOAc and washed with a 5% NaHCO_3_ solution to isolate pure methyl ester **11** (Scheme 1). A quantitative yield was obtained in only 60 min of residence time at 70 °C (Table 1, entry 2).

**Table 1:** Flow Synthesis of the Vanillic Acid Methyl Ester

Entry	Temperature (°C)	Residence time (min)	Isolated yield (%)^a^
1	65	90	65
2	70	60	quant.
3	70	30	70

a Isolated yields obtained after the following workup procedure: (i) solvent evaporation under reduced pressure, (ii) dissolution of the crude product in EtOAc, (iii) washing of the organic phase with a 5% NaHCO_3_ solution, (iv) drying over Na_2_SO_4_ and solvent evaporation under reduced pressure.

**Scheme 1:**
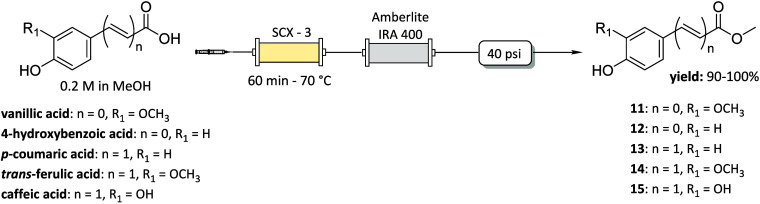
Flow Fischer Esterification and In-Line Purification

To avoid the manual workup procedure, an in-line purification was developed. The solution, containing the desired methyl ester **11**, after exiting from the SCX-3 packed bed reactor (PBR), was directed into a second glass column reactor containing Amberlite IRA 400­(OH^–^) as scavenger to catch the possibly unreacted vanillic acid (Scheme 1). This scavenger proved effective in trapping residual phenolic acid, reducing the time and manual handling associated with the traditional aqueous workup. MeOH was evaporated under reduced pressure and recovered. Afterward, the developed protocol was successfully applied to the synthesis of the desired methyl esters **12**–**15** that were recovered in very high yields (90→99%). Using a PBR with a volume of 2.4 mL (600 mg of SCX-3), esters **11**–**15** were produced in multigram quantity, in 24 h (1.5–2.2 g/day). Moreover, MeOH was recovered by evaporation and was reused.

For comparative purposes, the Fischer esterification reaction of vanillic acid was performed in batch mode using concentrated sulfuric acid in MeOH at reflux for 18 h. Following the workup procedure (for details see the Experimental Section), the methyl ester **11** was successfully isolated with an excellent yield (i.e., 95%). Despite the comparable yields between the batch and flow protocols, the batch process required a prolonged reaction time, the use of concentrated sulfuric acid, and extensive workup steps. These factors underscore the benefits of adopting flow chemistry, which offers a more efficient, automatized, and streamlined approach.

The synthesis of the desired amides was performed starting from the synthesized esters and a primary amine as nucleophile (i.e., 2-phenylethylamine and tyramine), using immobilized lipase B from *Candida antarctica* (imm-CaLB) as a biocatalyst in the presence of molecular sieves (4 Å). Initially, the reaction was investigated in batch using methyl ester **11** as the substrate and 2-phenylethylamine (PEA) as the nucleophile. *tert*-Amyl alcohol, a polar non-nucleophilic solvent classified as sustainable and green, was selected since it had already been proved capable to keep unaltered the catalytic activity of immobilized CaLB.^43,44^ The reaction was stirred at 80 °C for 24 h, but no product formation was observed. Therefore, different solvents (i.e., *i*-PrOH, CPME, *tert*-butyl methyl ether, toluene), temperature (45–80 °C), and reaction time (24–72 h) were screened. Only using toluene, product formation was observed. Thus, the biotransformation was performed using 3 equiv of PEA in toluene at 65 °C for 24 h, as longer times did not lead to an increase in conversion. Exploiting these conditions, after workup and purification, the final compound **1** was isolated in moderate yield (30%). Starting from this result, we focused on designing and developing an efficient flow setup to increase productivity and reduce reaction time. A stock solution containing the ester **11** as model starting material and PEA (2 equiv), as model nucleophile, in toluene was pumped through a glass column reactor packed with a mixture of immobilized CaLB and molecular sieves (4 Å) (1:1% w/w). Different experimental conditions were investigated to identify the best reaction conditions in the studied intervals and to achieve the highest conversion of compound **1**, as determined by HPLC analysis (Table 2).

**Table 2:** Optimization of the Flow Biocatalyzed Amidation Reaction Using Methyl Ester **11** as Substrate and PEA as Nucleophile^a^

Entry	Temperature (°C)	Residence time (min)	Molar ratio (ester 11/PEA)	Conversion (%)^b^
**1**	60	65	1:2	38
**2**	80	45	1:2	30
**3**	80	45	1:3	26
**4**	80	45	1:5	24
**5**	80	65	1:2	50
**6**	80	85	1:2	40
**7**	80	45	1:1.5	20

a Solutions in toluene. Concentration of compound **11**: 0.12 M.

b Molar conversion determined by HPLC analysis. The percentage of conversion was calculated on the basis of the depletion of compound **11** and monitoring the formation of the product **1**: Conversion [%] = [product area × *cf*/(product area × *cf* + compound **11** area)] × 100. According to the different absorbances of compounds **1** and **11** at 254 nm, a correction factor (*cf*) of 0.82 was used.

According to the HPLC analyses, the highest conversion achieved was 50% (Table 1, entry 5). An increase of the residence time or the equivalents of PEA was not beneficial to improve conversion (Table 2, entries 3, 4, 6).

Therefore, the desired compounds **1**–**10** were synthesized exploiting the conditions reported in Table 2, entry 5 (Scheme 2). For the synthesis of compounds **9** and **10**, the reactions were performed in the dark due to the presence of the catechol moiety. Moreover, due to solubility issues, the biotransformations starting from compounds **12** and **15**, as well as all the reactions using tyramine as nucleophile, were carried out in suspension exploiting a second peristaltic pump as oscillator and washing the PBR with *tert*-amyl alcohol at the end of the reaction. Compounds **1**–**10** were isolated in moderate to good yields after column chromatography.

**Scheme 2:**
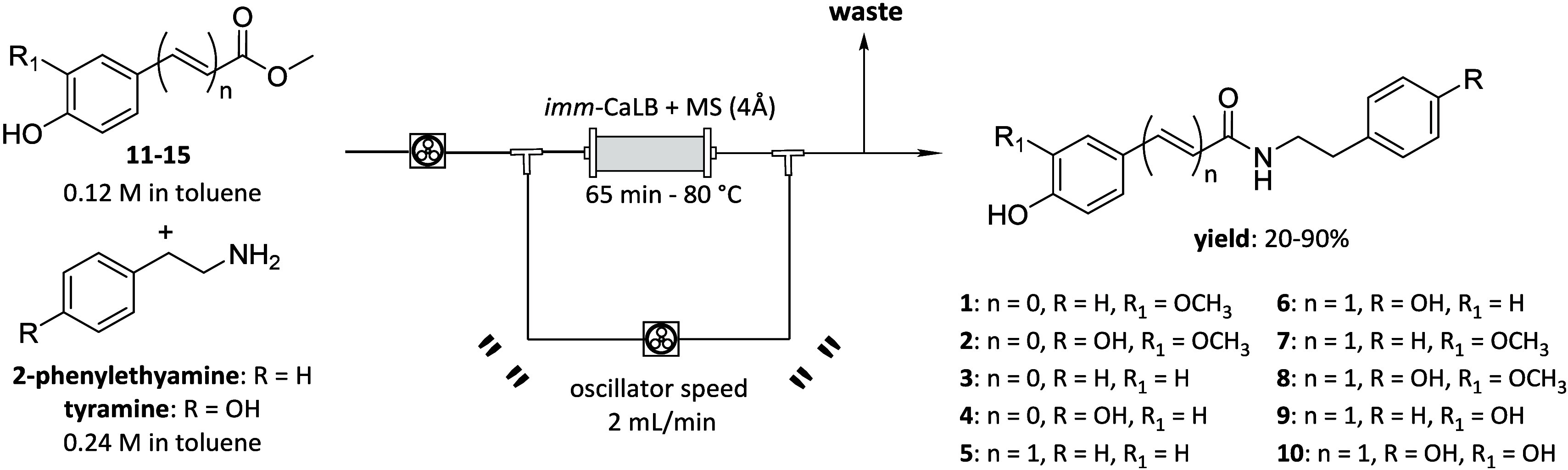
Biocatalyzed Flow Synthesis of Amides **1**–**10**

Given that phenolic compounds can serve as free radical scavengers by donating a hydrogen radical to form aryloxy radicals, the radical scavenging efficiency of compounds **1**–**10** at a concentration of 0.1 mM, using a 1,1-diphenyl-2-picrylhydrazyl (DPPH) radical scavenging assay (Table 3), was evaluated.^45^ Concerning compounds **1**–**8**, the tyramine derivatives **2**, **4**, **6**, and **8** showed an almost 2-fold higher percentage of inhibition compared to the PEA derivatives, possibly due to the presence of an additional phenolic moiety. Compound **10**, bearing a catechol moiety, was tested at a lower concentration of 0.01 mM, still showing a notable inhibition of 37%.

**Table 3:** Investigation of Free Radical Scavenging Activity of Compounds **1**–**10** ^a^

Compound	Concentration (mM)	% inhibition
**1**	0.1	30
**2**	0.1	63
**3**	0.1	28
**4**	0.1	59
**5**	0.1	34
**6**	0.1	66
**7**	0.1	29
**8**	0.1	67
**9**	0.1	63
**10**	0.01	37

a DPPH assays were conducted in duplicate. The results are expressed as means, and errors are within 10%.

In addition, recent studies have demonstrated that riparins and HCAAs exhibit significant antimicrobial properties, effectively inhibiting the growth of various bacterial strains.^46^ Moreover, these compounds have shown promising antileishmanial activity, making them potential candidates for the development of new treatments against Leishmania infections.^12–14^ Therefore, compounds **1**–**10** were investigated as antimicrobial agents, particularly as antibacterials in a panel of Gram-negative and Gram-positive bacteria (Table 4) and as antileishmanial agents (Table S1).

**Table 4:** Minimum Inhibitory Concentrations (MIC) of Compounds **1**–**10** ^b^

Compound	*P. aeruginosa* μg/mL (mM)	*S*. *aureus* μg/mL (mM)	*E*. *coli* μg/mL (mM)	*S*. *enterica*^a^ μg/mL (mM)
**1**	64 (0.24)	64 (0.24)	128 (0.48)	128 (0.48)
**2**	32 (0.11)	128 (0.44)	128 (0.44)	128 (0.44)
**3**	64 (0.27)	64 (0.27)	128 (0.54)	128 (0.54)
**4**	32 (0.12)	128 (0.48)	128 (0.48)	128 (0.48)
**5**	32 (0.12)	128 (0.48)	128 (0.48)	128 (0.48)
**6**	64 (0.23)	128 (0.46)	128 (0.46)	128 (0.46)
**7**	64 (0.24)	64 (0.24)	128 (0.48)	128 (0.48)
**8**	32 (0.10)	128 (0.40)	128 (0.40)	128 (0.40)
**9**	64 (0.22)	32 (0.11)	128 (0.44)	128 (0.44)
**10**	64 (0.22)	128 (0.44)	128 (0.44)	128 (0.44)

a The assays were performed in triplicate.

b *S. enterica* subsp. *enterica* ser. Enteritidis.

All compounds exhibited similar MIC values (128 μg/mL) against *E. coli* and *S. enterica*, suggesting a consistent level of effectiveness across these strains. Compounds **2**, **4**, **5**, and **8** showed the lowest MIC values against *P. aeruginosa* (32 μg/mL), 4-fold lower compared to the MIC against the other strains. Compound **9** is more effective against *S. aureus* (32 μg/mL) than the other compounds, which generally have higher MIC values (64–128 μg/mL). Finally, a phenotypic assay on *L. tropica* and *L. infantum* promastigotes was performed using the MTT (3-(4,5-dimethylthiazol-2-yl)-2,5-diphenyltetrazolium bromide) method and amphotericin-B as a positive control (Table S1). Only compound **7** displayed a weak antileishmanial activity toward *L. infantum* and *L. tropica* (IC_50_ = 17 and 15 μM, respectively). The cLogP values of compounds **1**–**10**, ranging from 2.14 to 3.53 (Table S1), indicate a balance between hydrophilicity and lipophilicity, which is crucial for the effective interaction with microbial cell membranes. Compounds with cLogP values within this range are likely to have good membrane permeability, enhancing their ability to penetrate microbial cells and exert their antimicrobial effects. Additionally, these values are within the optimal range for drug-likeness, suggesting that the compounds could be promising candidates for further development as antimicrobial agents.

## Conclusions

The development of new methods for creating amide bonds is essential in the pharmaceutical and fine chemical fields. These innovative approaches enable the creation of more efficient and sustainable synthetic pathways that lead to the production of a wide range of compounds. This study demonstrates the successful development of a two-step chemo-enzymatic flow method for the esterification and amidation of phenolic acids, yielding esters and amides with high efficiency and purity. First, a series of methyl esters was prepared by exploiting the continuous Fischer esterification of selected natural phenolic acids in the presence of a resin with strong acid groups as the catalyst. Our approach allowed us to significantly improve safety and simplify workup procedures, when compared to the batch reaction. Afterward, the newly synthesized derivatives were submitted to the biocatalyzed flow reaction with selected primary amines, in the presence of immobilized *Candida antarctica* lipase as biocatalyst. This method was effectively applied to synthesize riparin derivatives and HCAAs, which were subsequently evaluated for their radical scavenging, antibacterial, and antileishmanial properties. The results highlight the potential of enzymatic approaches in producing pharmacologically active compounds with a variety of biological activities. Despite some challenges in optimizing the flow conditions for amidation, the developed protocol offers a promising alternative to traditional chemical methods, emphasizing the importance of sustainable and efficient synthetic routes in pharmaceutical and materials science.

## Experimental Section

### General Experimental Procedures

Reagents and solvents were obtained from commercial suppliers and used without further purification. Immobilized lipase B from *Candida antarctica* was purchased from Merck. NMR spectra were recorded on a Varian Gemini 300 MHz spectrometer using the residual signal of the deuterated solvent as an internal standard. ^1^H chemical shifts (δ) are expressed in ppm, and coupling constants (*J*) in hertz (Hz). HPLC analyses were performed using a Waters 1525 Binary HPLC pump equipped with a Waters 2489 UV–vis detector (Waters, Milford, MA, USA) and a Waters C18 μBondapack column (10 μm, 125 Å, 254 nm). Continuous flow reactions were performed using a Series E flow reactor (Vaportec) or Asia Flow Chemistry syringe pumps (Syrris) equipped with Omnifit glass columns (6.6 mm i.d. × 100 mm length or 10 mm i.d. × 100 mm length). Pressure was controlled using back-pressure regulators. TLC analyses were performed on commercial silica gel 60 F_254_ aluminum sheets; spots were further evidenced by spraying with a dilute alkaline solution of KMnO_4_ or ninhydrin. The DPPH radical-scavenging assay (Bioquochem, Asturie, Spain) was performed using a spectrophotometer (Eppendorf, Milan, Italy).

#### HPLC Analyses

Injection volume: 20 μL. Flow rate: 1.0 mL min^–1^. Mobile phase: (A) H_2_O; (B) MeOH; gradient conditions: 0–7 min 70% (A)/30% (B), 7–19 min 60% (A)/40% (B); 19–22 min 50% (A)/50% (B); 22–30 min 70% (A)/30% (B). Retention times (*t*_R_): PEA = 4.7 min; methyl 3-hydroxy-4-methoxybenzoate (**11**) = 8.2 min; 4-hydroxy-3-methoxy-*N*-phenethylbenzamide (**1**) = 11.9 min.

#### Batch Synthesis of Methyl 3-Hydroxy-4-methoxybenzoate, **11**

To a solution of vanillic acid (1 equiv) in MeOH (0.2 M) was added concentrated H_2_SO_4_ (0.01 equiv). The reaction mixture was refluxed for 18 h, then cooled to room temperature and quenched with water. A 5% aqueous solution of NaHCO_3_ was added to reach pH = 8, and MeOH was evaporated under reduced pressure. The resulting mixture was extracted with EtOAc. The organic phase was washed with brine, dried over Na_2_SO_4_, and evaporated under reduced pressure to give pure methyl ester **11** in 95% yield.

#### Flow Synthesis of Methyl Esters **11**–**15**

A stock solution of the proper phenolic acid in MeOH (0.2 M) was pumped by an Asia syringe pump through an Omnifit column reactor (*V* = 2.4 mL; residence time = 60 min) packed with 0.6 g of SCX-3 (Silia*Bond* Tosic Acid) heated at 70 °C. MeOH was used as flow stream. The exiting solution was flowed through an unpowered glass column reactor (*V* = 0.9 mL; residence time = 22 min) packed with 0.2 g of Amberlite IRA 400 (OH−). The system was pressurized at 20 psi, and the exiting solution was collected in a round-bottomed flask. The solvent was evaporated under reduced pressure, and the desired products were successfully isolated without any further purification.

##### Methyl 4-hydroxy-3-methoxybenzoate (**11**)

Yield: 90%; yellow oil; *R*_*f*_: 0.61 (cyclohexane/EtOAc, 1:1); ^1^H NMR (300 MHz, methanol-*d*_4_) δ 7.55–7.52 (m, 2H), 6.81 (d, *J* = 9.1 Hz, 1H), 3.88 (s, 3H), 3.84 (s, 3H).

##### Methyl 4-hydroxybenzoate (**12**)

Yield: 95%; white solid; *R*_f_: 0.60 (cyclohexane/EtOAc, 1:1); ^1^H NMR (300 MHz, methanol-*d*_4_) δ 7.87 (d, *J* = 8.8 Hz, 2H), 6.80 (d, *J* = 8.8 Hz, 2H), 3.85 (s, 3H).

##### Methyl (*E*)-3-(4-hydroxyphenyl)­acrylate (**13**)

Yield: 92%; white solid; *R*_*f*_: 0.62 (cyclohexane/EtOAc, 1:1); ^1^H NMR (300 MHz, methanol-*d*_4_) δ 7.60 (d, *J* = 15.1 Hz, 1H), 7.44 (d, *J* = 9.1 Hz, 2H), 6.80 (d, *J* = 9.1 Hz, 2H), 6.31 (d, *J* = 15.1 Hz, 1H), 3.75 (s, 3H).

##### Methyl (*E*)-3-(4-hydroxy-3-methoxyphenyl)­acrylate (**14**)

Yield: 94%; yellow oil; *R*_*f*_: 0.40 (cyclohexane/EtOAc, 6:4); ^1^H NMR (300 MHz, methanol-*d*_4_) δ 7.60 (d, *J* = 15.0 Hz, 1H), 7.17 (d, *J* = 3.1 Hz, 1H), 7.06 (dd, *J* = 6.0, 3.1 Hz, 1H), 6.80 (d, *J* = 6.0 Hz, 1H), 6.35 (d, *J* = 15.0 Hz, 1H), 3.88 (s, 3H), 3.76 (s, 3H).

##### Methyl (*E*)-3-(3,4-dihydroxyphenyl)­acrylate (**15**)

Yield: 96%; white solid; *R*_*f*_: 0.27 (DCM/MeOH, 95:5); ^1^H NMR (300 MHz, acetone-*d*_6_) δ 8.45–8.10 (bs, 2H), 7.53 (d, *J* = 15.2 Hz, 1H), 7.15 (d, *J* = 3.1 Hz, 1H), 7.04 (dd, *J* = 9.1, 3.1 Hz, 1H), 6.86 (d, *J* = 9.1 Hz, 1H), 6.28 (d, *J* = 15.2 Hz, 1H), 3.71 (s, 3H).

#### Batch Synthesis of 4-Hydroxy-3-methoxy-*N*-phenethylbenzamide (**1**)

To a mixture of methyl ester **11** in toluene (1 equiv, 0.12 M) and 2-phenylethylamine (3 equiv) were added immobilized CaLB and molecular sieves 4 Å (1:1% w/w). The mixture was left under magnetic stirring, at 65 °C, for 24 h. After this time, the mixture was filtered under vacuum and the solvent was evaporated under reduced pressure. The obtained crude product was purified by silica flash column chromatography (eluent: cyclohexane/EtOAc from 8:2 to 7:3) to yield the desired amide **1** in 30% yield.

#### Biocatalyzed Continuous Flow Synthesis of Compounds **1**–**10**

A solution of the methyl ester **11**–**15** (1 equiv, 0.12 M) and 2-phenylethylamine or tyramine (2 equiv, 0.24 M) in toluene (10 mL) was prepared. The solution was pumped by a peristaltic pump through an Omnifit column reactor packed with a mixture of immobilized CaLB (2.5 g) and molecular sieves (4 Å, 2.5 g, total volume = 9.0 mL, residence time = 65 min). Toluene was used as flow stream, and the temperature was set at 80 °C. For substrates **12**, **15**, and all tyramine derivatives, a peristaltic pump was set as oscillator with an oscillation rate of 2.0 mL min^–1^. For the preparation of compounds **2**, **4**, **6**, **8**, and **10**, a second flow stream of *tert*-amyl alcohol was used to wash the reactor and to recover all the reaction crude mixture. The exiting solution was collected in a round-bottomed flask, and the solvent was evaporated under reduced pressure. The crude products were purified by silica gel column chromatography.

##### 4-Hydroxy-3-methoxy-*N*-phenethylbenzamide (**1**)

Eluent for column chromatography: cyclohexane/EtOAc from 8:2 to 7:3. Yield: 47%; white solid; *R*_*f*_: 0.36 (cyclohexane/EtOAc, 1:1); ^1^H NMR (300 MHz, methanol-*d*_4_) δ 7.38 (d, *J* = 3.0 Hz, 1H), 7.35–7.15 (m, 6H), 6.81 (d, *J* = 9.0 Hz, 1H), 3.88 (s, 3H), 3.56 (t, *J* = 9.1 Hz, 2H), 2.90 (t, *J* = 9.1 Hz, 2H); ^13^C NMR (75 MHz, methanol-*d*_4_) δ 168.5, 149.7, 147.3, 139.2, 128.4, 128.0, 125.9, 125.5, 120.5, 114.4, 110.5, 54.9, 41.2, 35.2; HR-MS calcd for C_16_H_17_NO_3_Na [M + Na]^+^ 294.1106; found 294.1106.

##### 4-Hydroxy-*N*-(4-hydroxyphenethyl)-3-methoxybenzamide (**2**)

Eluent for column chromatography: cyclohexane/EtOAc from 7:3 to 6:4. Yield: 75%; white solid; *R*_*f*_: 0.20 (DCM/MeOH, 95:5); ^1^H NMR (300 MHz, methanol-*d*_4_) δ 7.37 (d, *J* = 3.1 Hz, 1H), 7.29 (dd, *J* = 9.2, 3.1 Hz, 1H), 7.04 (d, *J* = 9.2 Hz, 2H), 6.81 (d, *J* = 9.2 Hz, 1H), 6.71 (d, *J* = 9.2 Hz, 2H), 3.86 (s, 3H), 3.50 (t, *J* = 9.0 Hz, 2H), 2.78 (t, *J* = 9.0 Hz, 2H); ^13^C NMR (75 MHz, methanol-*d*_4_) δ 168.9, 155.4, 149.7, 147.3, 130.0, 129.4, 125.6, 120.4, 114.8, 114.4, 110.4, 54.9, 41.5, 34.4; HR-MS calcd for C_16_H_17_NO_4_Na [M + Na]^+^ 310.1055; found 310.1054.

##### 4-Hydroxy-*N*-phenethylbenzamide (**3**)

Eluent for column chromatography: cyclohexane/EtOAc, 7:3. Yield: 69%; yellow solid; *R*_*f*_: 0.32 (cyclohexane/EtOAc, 1:1); ^1^H NMR (300 MHz, methanol-*d*_4_) δ 7.64 (d, *J* = 9.2 Hz, 2H), 7.26–7.15 (m, 5H), 6.80 (d, *J* = 9.2 Hz, 2H), 3.55 (t, *J* = 9.2 Hz, 2H), 2.89 (t, *J* = 9.2 Hz, 2H); ^13^C NMR (75 MHz, methanol-*d*_4_) δ: 168.6, 160.6, 139.2, 128.8, 128.4, 128.0, 125.9, 125.1, 114.7, 41.2, 35.3; HR-MS calcd for C_15_H_15_NO_2_Na [M + Na]^+^ 264.1000; found 264.0995.

##### 4-Hydroxy-*N*-(4-hydroxyphenethyl)­benzamide (**4**)

Eluent for column chromatography: cyclohexane/EtOAc from 8:2 to 3:7. Yield: 75%; white solid; *R*_*f*_ 0.28 (DCM/MeOH 9:1); ^1^H NMR (300 MHz, methanol-*d*_4_) δ 7.64 (d, *J* = 9.0 Hz, 2H), 7.05 (d, *J* = 9.0 Hz, 2H), 6.79 (d, *J* = 9.0 Hz, 2H), 6.70 (d, *J* = 9.0 Hz, 2H), 3.49 (t, *J* = 9.3 Hz, 2H), 2.78 (t, *J* = 9.3 Hz, 2H); ^13^C NMR (75 MHz, methanol-*d*_4_) δ 168.6, 160.5, 155.4, 130.0, 129.4, 128.7, 125.1, 114.8, 114.6, 41.5, 34.4; HR-MS calcd for C_15_H_15_NO_3_Na [M + Na]^+^ 280.0950; found 280.0948.

##### (*E*)-3-(4-Hydroxyphenyl)-*N*-phenethylacrylamide (**5**)

Eluent for column chromatography: cyclohexane/EtOAc from 7:3 to 1:1. Yield: 60%; yellow solid; *R*_*f*_ 0.36 (cyclohexane/EtOAc 1:1); ^1^H NMR (300 MHz, methanol-*d*_4_) δ 7.45 (d, *J* = 15.1 Hz, 1H), 7.37 (d, *J* = 9.2 Hz, 2H), 7.26–7.16 (m, 5H), 6.78 (d, *J* = 9.2 Hz, 2H), 6.38 (d, *J* = 15.1 Hz, 1H), 3.49 (t, *J* = 9.0 Hz, 2H), 2.82 (t, *J* = 9.0 Hz, 2H); ^13^C NMR (75 MHz, methanol-*d*_4_) δ 167.8, 159.1, 140.4, 139.0, 129.2, 128.4, 128.1, 126.2, 125.9, 117.0, 115.3, 40.8, 35.2; HR-MS calcd for C_17_H_17_NO_2_Na [M + Na]^+^ 290.1157; found 290.1157.

##### (*E*)-*N*-(4-Hydroxyphenethyl)-3-(4-hydroxyphenyl)­acrylamide (**6**)

The crude product was washed with diluted HCl (0.1 M) to remove unreacted tyramine. The organic layer was washed with brine, dried over Na_2_SO_4_, and filtered, and the solvent was evaporated under reduced pressure. Yield: quantitative; white solid; *R*_*f*_ 0.30 (DCM/MeOH, 9:1); ^1^H NMR (300 MHz, methanol-*d*_4_) δ 7.45 (d, *J* = 15.2 Hz, 1H), 7.35 (d, *J* = 9.0 Hz, 2H), 7.04 (d, *J* = 9.0 Hz, 2H), 6.78 (d, *J* = 9.0 Hz, 2H), 6.71 (d, *J* = 9.0 Hz, 2H), 6.38 (d, *J* = 15.2 Hz, 1H), 3.45 (t, *J* = 9.2 Hz, 2H), 2.74 (t, *J* = 9.2 Hz, 2H); ^13^C NMR (75 MHz, methanol-*d*_4_) δ 167.8, 159.0, 155.4, 140.3, 129.8, 129.3, 129.1, 126.2, 117.0, 115.3, 114.8, 41.1, 34.4; HR-MS calcd for C_17_H_17_NO_3_Na [M + Na]^+^ 306.1106; found 306.1109.

##### (*E*)-3-(4-Hydroxy-3-methoxyphenyl)-*N*-phenethylacrylamide (**7**)

Eluent for column chromatography: cyclohexane/EtOAc from 8:2 to 7:3. Yield: 50%; yellow solid; *R*_*f*_ 0.39 (cyclohexane/EtOAc, 7:3); ^1^H NMR (300 MHz, methanol-*d*_4_) δ 7.43 (d, *J* = 15.1 Hz, 1H), 7.29–7.18 (m, 5H), 7.10 (d, *J* = 3.2 Hz, 1H), 7.01 (dd, *J* = 9.2, 3.2 Hz, 1H), 6.78 (d, *J* = 9.2 Hz, 1H), 6.39 (d, *J* = 15.1 Hz, 1H), 3.87 (s, 3H), 3.51 (t, *J* = 9.2 Hz, 2H), 2.84 (t, *J* = 9.2 Hz, 2H); ^13^C NMR (75 MHz, methanol-*d*_4_) δ 167.7, 148.4, 147.8, 140.6, 139.1, 128.3, 128.0, 126.8, 125.9, 121.7, 117.2, 115.0, 110.0, 54.9, 40.8, 35.2; HR-MS calcd for C_18_H_19_NO_3_Na [M + Na]^+^ 320.1263; found 320.1264.

##### (*E*)-3-(4-Hydroxy-3-methoxyphenyl)-*N*-(4-hydroxyphenethyl)­acrylamide (**8**)

Eluent for column chromatography: cyclohexane/EtOAc from 6:4 to 1:1. Yield: 47%; yellow solid; *R*_*f*_ 0.28 (DCM/MeOH, 95:5); ^1^H NMR (300 MHz, methanol-*d*_4_) δ 7.43 (d, *J* = 15.1 Hz, 1H), 7.07 (d, *J* = 3.1 Hz, 1H), 7.04–6.90 (m, 3H), 6.78 (d, *J* = 9.2 Hz, 1H), 6.71 (d, *J* = 9.2 Hz, 2H), 6.40 (d, *J* = 15.1 Hz, 1H), 3.80 (s, 3H), 3.45 (t, *J* = 9.1 Hz, 2H), 2.73 (t, *J* = 9.1 Hz, 2H); ^13^C NMR (75 MHz, methanol-*d*_4_) δ 167.8, 155.5, 148.4, 147.8, 140.7, 129.9, 129.4, 126.8, 121.9, 117.3, 115.1, 114.9, 110.1, 55.0, 41.2, 34.4; HR-MS calcd for C_18_H_19_NO_4_Na [M + Na]^+^ 336.1212; found 336.1211.

##### (*E*)-3-(3,4-Dihydroxyphenyl)-*N*-phenethylacrylamide (**9**)

Eluent for column chromatography: cyclohexane/EtOAc, 1:1. Yield: 20%; yellow solid; *R*_*f*_ 0.28 (cyclohexane/EtOAc, 1:1); ^1^H NMR (300 MHz, methanol-*d*_4_) δ 7.37 (d, *J* = 15.1 Hz, 1H), 7.28–7.14 (m, 5H), 6.98 (d, *J* = 3.2 Hz, 1H), 6.88 (dd, *J* = 6.0 Hz, 3.2 Hz, 1H), 6.75 (d, *J* = 6.0 Hz, 1H), 6.31 (d, *J* = 15.1 Hz, 1H), 3.50 (t, *J* = 9.1 Hz, 2H), 2.84 (t, *J* = 9.1 Hz, 2H); ^13^C NMR (75 MHz, methanol-*d*_4_) δ 167.8, 147.4, 145.4, 140.8, 139.1, 128.6, 128.4, 128.0, 125.9, 120.6, 116.8, 115.0, 113.5, 40.8, 35.2; HR-MS calcd for C_17_H_17_NO_3_Na [M + Na]^+^ 306.1106; found 306.1107.

##### (*E*)-3-(3,4-Dihydroxyphenyl)-*N*-(4-hydroxyphenethyl)­acrylamide (**10**)

Eluent for column chromatography: cyclohexane/EtOAc from 6:4 to 1:9. Yield: 20%; white solid; *R*_*f*_: 0.34 (DCM/MeOH, 9:1); ^1^H NMR (300 MHz, methanol-*d*_4_) δ 7.38 (d, *J* = 15.0 Hz, 1H), 7.04 (d, *J* = 9.1 Hz, 2H), 6.99 (d, *J* = 3.1 Hz, 1H), 6.89 (dd, *J* = 9.1, 3.1 Hz, 1H), 6.75 (d, *J* = 9.1 Hz, 1H), 6.71 (d, *J* = 9.1 Hz, 2H), 6.32 (d, *J* = 15.0 Hz, 1H), 3.44 (t, *J* = 9.1 Hz, 2H), 2.73 (t, *J* = 9.1 Hz, 2H); ^13^C NMR (75 MHz, methanol-*d*_4_) δ 167.8, 155.4, 147.3, 145.2, 140.7, 129.8, 129.3, 126.8, 120.7, 116.9, 115.0, 114.8, 113.6, 41.1, 34.3; HR-MS calcd for C_17_H_17_NO_4_Na [M + Na]^+^ 322.1055; found 322.1056.

#### DPPH Radical-Scavenging Assay

Measurement of 1,1-diphenyl-2-picrylhydrazyl (DPPH^•^) radical-scavenging activity was performed using a commercial kit (Bioquochem, Asturie, Spain). Briefly, samples were appropriately dissolved in dimethyl sulfoxide (DMSO) and mixed with the DPPH solution provided by the kit. Trolox at different concentrations was used to build the standard curve. The antioxidant activity was determined by measuring absorbance at 517 nm by a spectrophotometer (Eppendorf, Milan, Italy) and calculating the corresponding percentage of inhibition following the guidelines of the provider. The assays were performed in independent duplicates. The results are expressed as means, and errors are within 10%.

#### Bacterial Strains and Culture Conditions

*Escherichia coli* ATCC 25922 (Ec), *Salmonella enterica* subsp. *enterica* ser*. Enteritidis* ISM 8324 (Se), *Pseudomonas aeruginosa* IMV 1 (Pa), and *Staphylococcus aureus* ATCC 6538 (Sa) were used for the evaluation of the antibacterial activity of the compounds. Stocks of the previously identified bacteria were thawed, and then they were streaked onto blood agar plates (tryptic soy agar + 5% sheep blood [Microbiol, Italy]) and incubated at 37 °C for 24 h under aerobic conditions to obtain isolated colonies to be used for the minimum inhibitory concentration (MIC) tests.

#### Determination of the Minimum Inhibitory Concentration

The MIC was determined using the microdilution assay according to the Clinical and Laboratory Standards Institute (CLSI) guidelines (2018, Performance Standards for Antimicrobial Susceptibility Testing, CLSI Approved Standard M100-S15, Clinical and Laboratory Standards Institute, Wayne, PA, USA). Briefly, all the previous identified strains were grown on tryptic soy agar (TSA, Oxoid, Milan, Italy), and 3 or 4 isolated colonies (depending on the size of the colonies) were suspended in sterile saline solution (9 g/L NaCl) to reach an initial concentration of 1.5 × 10^8^ CFU/mL (equivalent to 0.5 MacFarland standard). One hundred microliters of the 1:100 diluted cell suspensions was dispensed into each well of a 96-well microtiter plate. The strains were exposed to a 2-fold dilution series of each derivative (dissolved in DMSO). After incubation for 24 h at 37 °C aerobically, the MICs were determined as the lowest dilution of molecules able to inhibit visible bacterial growth. Positive strain control and negative control were tested for each plate. Assays were performed in triplicate.

#### Antileishmanial Activity Assay

Promastigote stage of *L. infantum* strain (MHOM/TN/80/IPT1, kindly provided by Dr. M. Gramiccia and Dr. T. Di Muccio, ISS, Roma) and *L. tropica* (MHOM/SY/2012/ISS3130) were cultured in RPMI 1640 medium (EuroClone) supplemented with 10% heat-inactivated fetal calf serum (EuroClone), 20 mM HEPES, and 2 mM l-glutamine at 24 °C. To estimate the 50% inhibitory concentration (IC_50_) toward *L. infantum* strain and *L. tropica* promastigotes, the MTT method was used, as previously reported.^47,48^

Compounds were dissolved in DMSO and then diluted with a medium to achieve the required concentrations. Drugs were placed in 96-well round-bottom microplates, and seven serial dilutions were made. Parasites were diluted in complete medium to 5 × 10^6^ parasites/mL, 100 μL of the suspension was seeded into the plates and incubated at 24 °C for 72 h, and then 20 μL of MTT solution (5 mg/mL) was added into each well for 3 h. The plates were then centrifuged at 1000*g* for 8 min at rt, the supernatants discarded, and the resulting pellets dissolved in 100 μL of lysing buffer consisting of 20% (w/v) of a solution of SDS (Sigma) and 40% of DMF (Merck) in H_2_O. The absorbance was measured spectrophotometrically at a test wavelength of 550 nm and a reference wavelength of 650 nm. The results are expressed as IC_50_, which is the dose of compound necessary to inhibit parasite growth by 50%; each IC_50_ value is the mean of separate experiments performed in duplicate.

## Supplementary Material



## Notes

^1^H NMR spectra for compounds **11**–**16**; ^1^H and ^13^C NMR and HRMS spectra of compounds **1**–**10**; antileishmanial activity of compounds **1**–**10** (PDF)
