# Supplementary material for: Two-Step Flow Amidation of Natural Phenolic Acids as Antiradical and Antimicrobial Agents
Source: J Nat Prod. 2025 Mar 31;88(5):1153–9. doi: 10.1021/acs.jnatprod.5c00131 (PMC12105028; doi:10.1021/acs.jnatprod.5c00131)
Supplement: Supplementary file 1 [file np5c00131_si_001.pdf]

SUPPORTING INFORMATION

TWO-STEP FLOW AMIDATION OF NATURAL PHENOLIC ACIDS AS ANTIRADICAL AND ANTIMICROBIAL AGENTS

Desirée Pecora[1], Anna M. Magni[1], Sara Vicinanza[1], Francesca Annunziata[2], Salvatore Princiotto[2], Silvia Donzella[2], Gabriele Meroni[3], Piera A. Martino[3], Nicoletta Basilico[3], Silvia Parapini[4], Paola Conti[1], Chiara Borsari[1], and Lucia Tamborini[1,]*

[1]Department of Pharmaceutical Sciences, University of Milan, via Mangiagalli 25, 20133 Milan, Italy; [2]Department of Food, Environmental and Nutritional Sciences, University of Milan, via Celoria 2, 20133 Milan, Italy; [3]Department of Biomedical, Surgical and Dental Sciences, One Health Unit, University of Milan, Via Pascal 36, 20133, Milan, Italy; [4]Department of Biomedical Sciences of Health, Via Pascal 36, 20133, Milan, Italy.

*Correspondence: lucia.tamborini@unimi.it; Tel.: +39 0250319367

Table of contents

^{1}H spectra of compounds **11-15** Page S2-S4

^{1}H, ^{13}C NMR and HRMS spectra of compounds **1-10** Page S5-S24

Table S1. Antileishmanial activity towards *L. infantum* and *L. tropica* and calculated LogP of compounds **1-10**. Page S25

Figure S1. ^{1}H NMR of compound **11**.

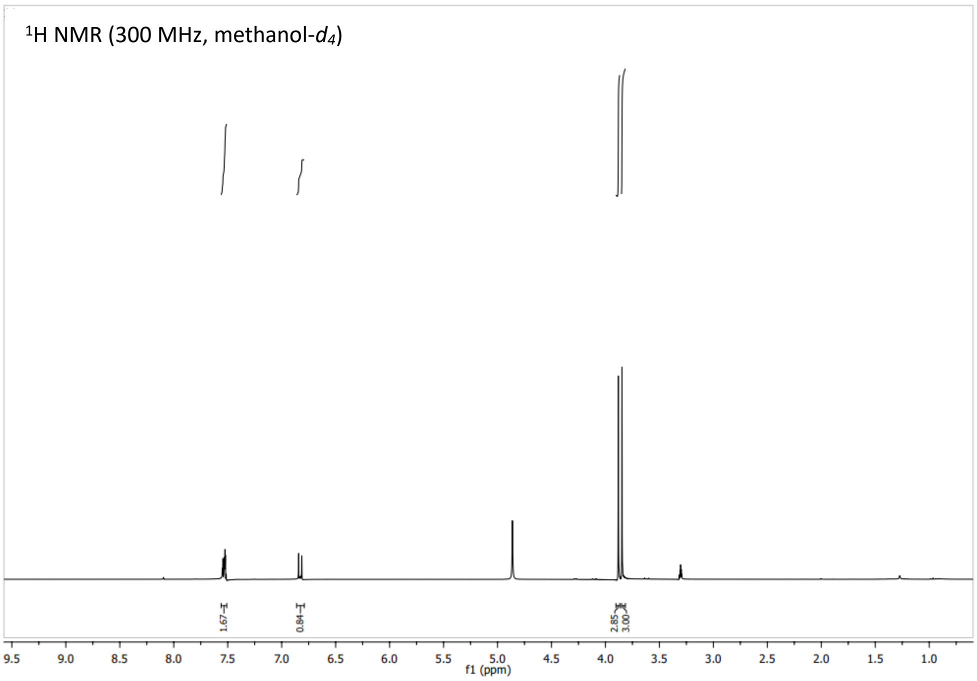

Figure S2. ^{1}H NMR of compound **12**.

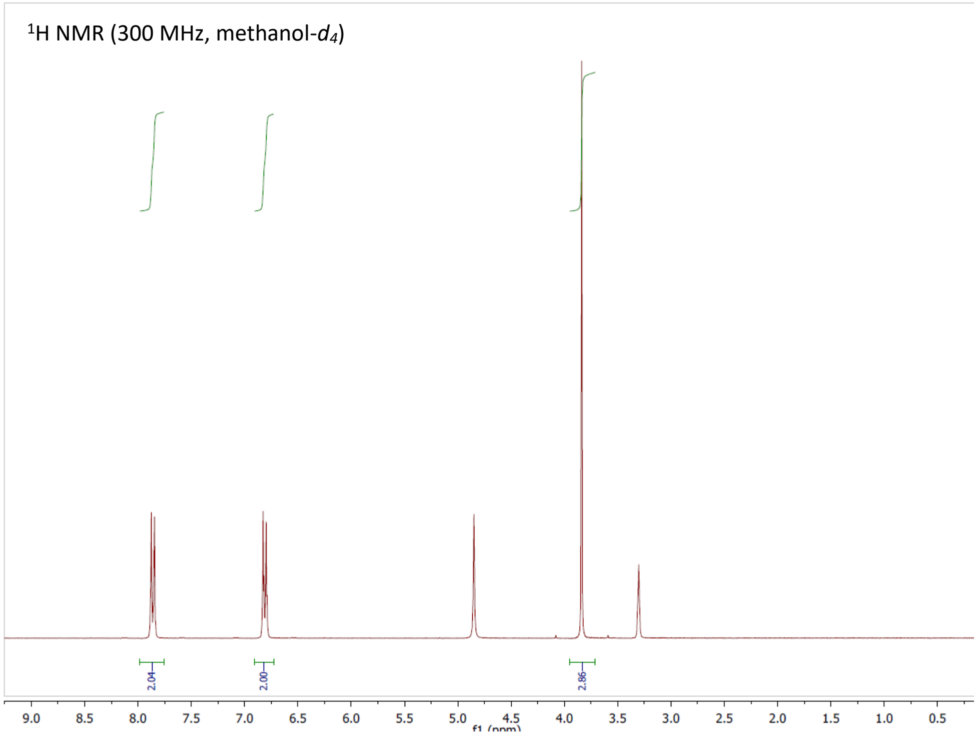

Figure S3. ^{1}H NMR of compound **13**.

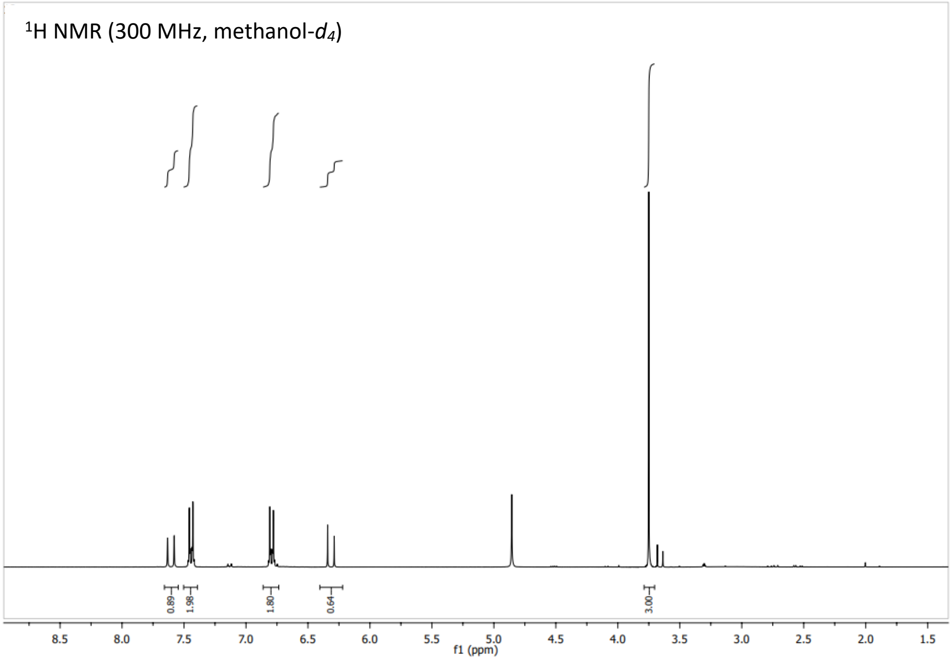

Figure S4. ^{1}H NMR of compound **14**.

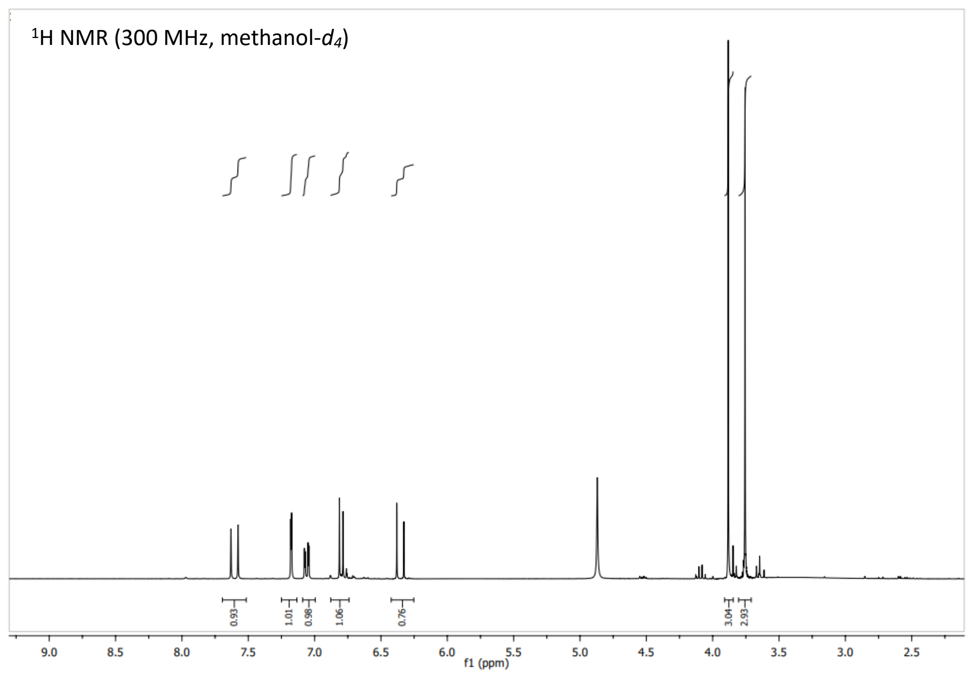

Figure S5. ^{1}H NMR of compound **15**.

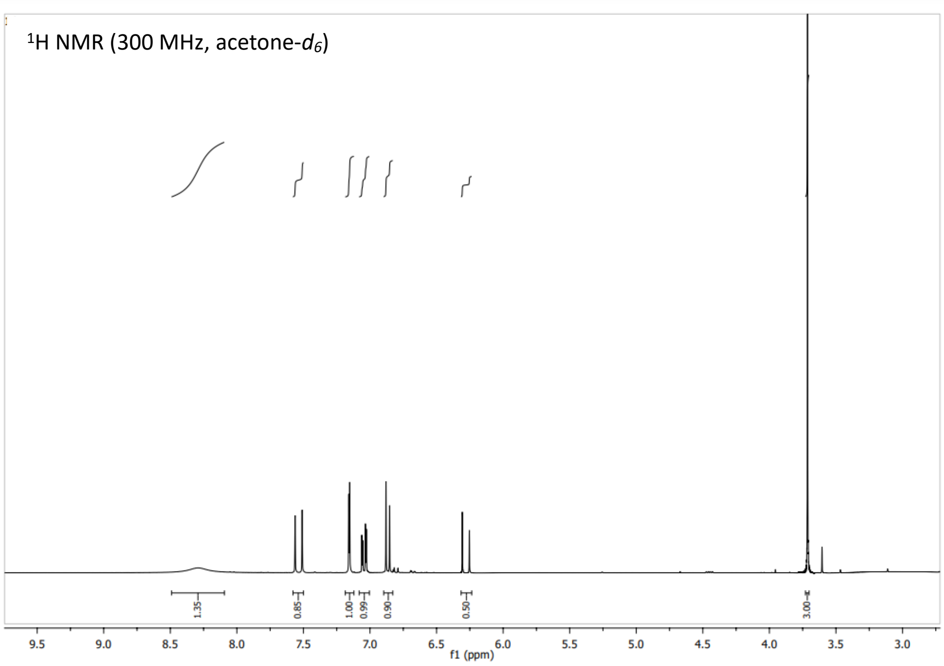

Figure S6. ^{1}H NMR of compound **1**.

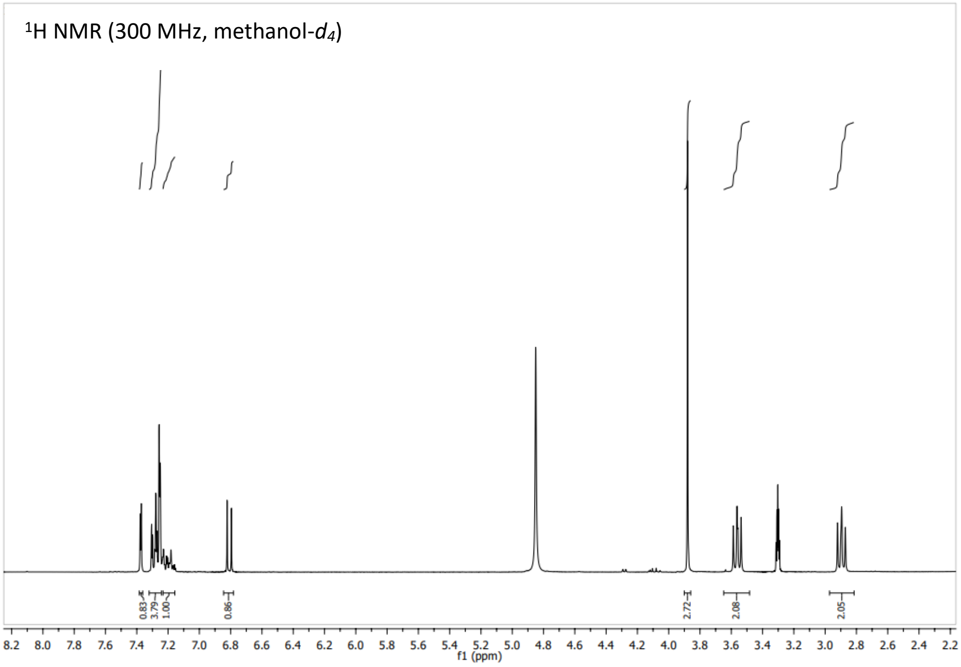

Figure S7. ^{13}C NMR of compound **1**.

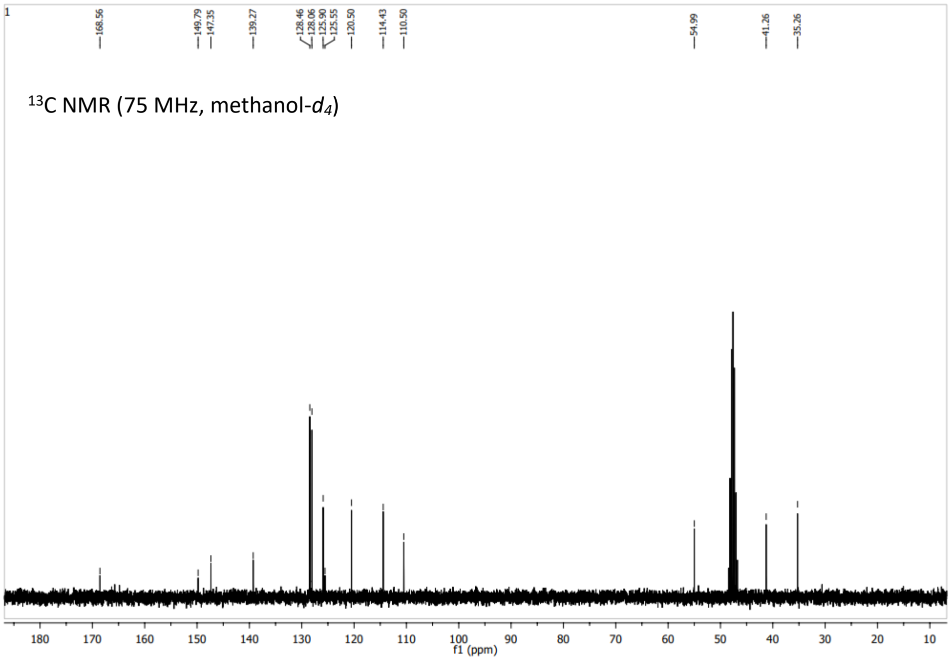

Figure S8. HRMS of compound **1**.

Elemental Composition Report

Page 1

Single Mass Analysis

Tolerance = 2.0 PPM / DBE: min = -5.0, max = 300.0
Element prediction: Off
Number of isotope peaks used for i-FIT = 5

Monoisotopic Mass, Even Electron Ions
2 formula(e) evaluated with 1 results within limits (all results (up to 1000) for each mass)
Elements Used:
C: 15-18 H: 14-20 N: 1-1 O: 2-4 Na: 0-1

AM1 30 (0.603) AM2 (Ar,40000.0,0.00,0.00); Cm (30:50)

1: TOF MS ES+
1.02e+007

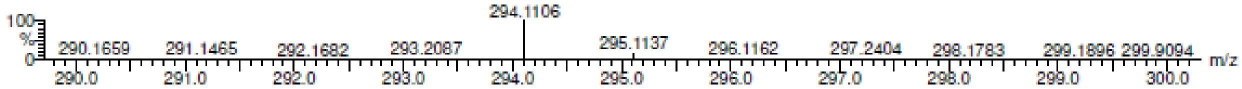

Minimum:				-5.0				
Maximum:		20.0	2.0	300.0				
Mass	Calc. Mass	mDa	PPM	DBE	i-FIT	Norm	Conf(%)	Formula
294.1106	294.1106	0.0	0.0	8.5	2710.3	n/a	n/a	C16 H17 N O3 Na

AM1 30 (0.603) AM2 (Ar,40000.0,0.00,0.00); Cm (30:50)

1: TOF MS ES+
1.02e7

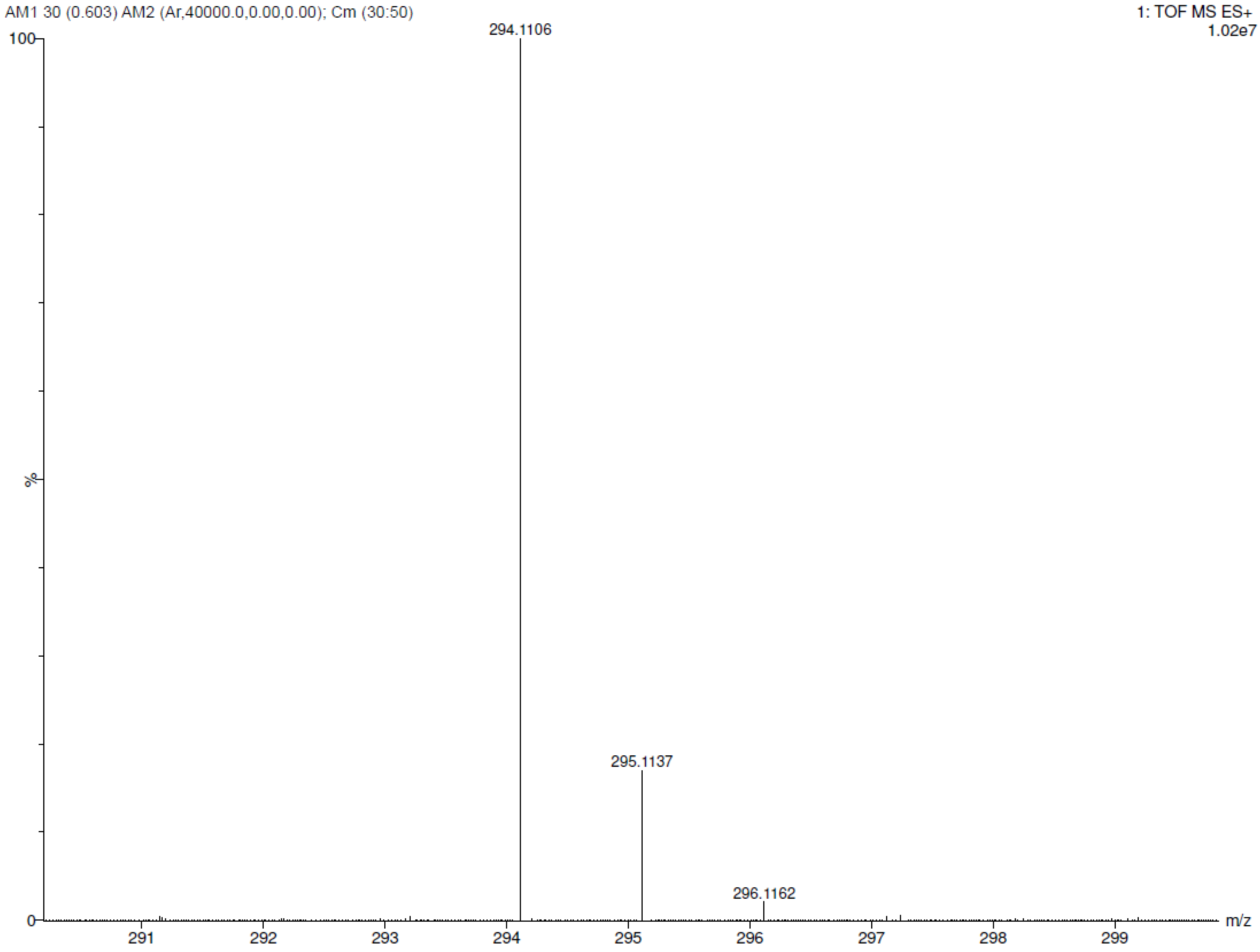

Figure S9. ^{1}H NMR of compound **2**.

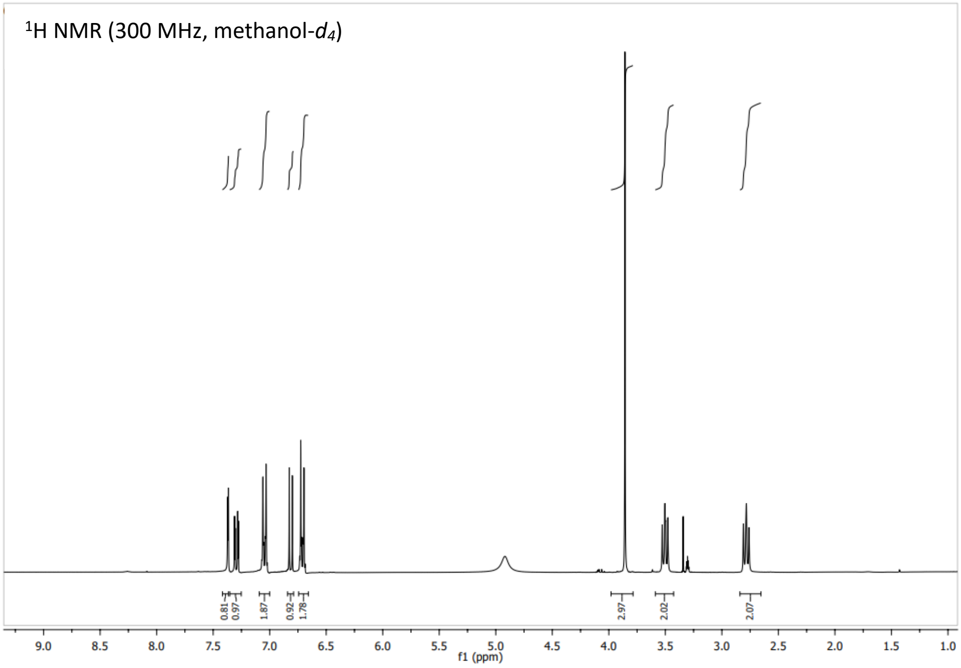

Figure S10. ^{13}C NMR of compound **2**.

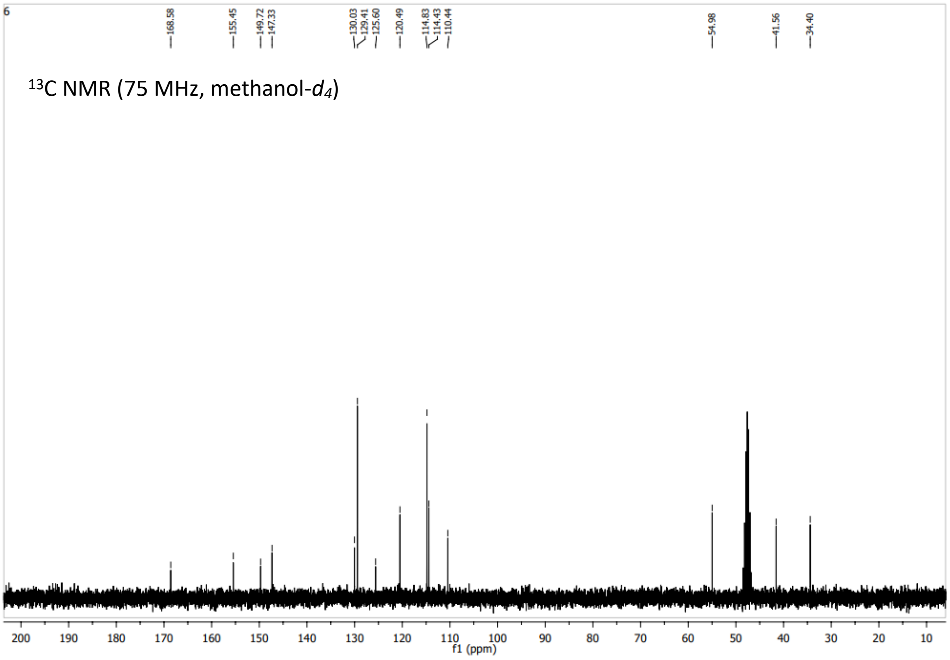

Figure S11. HRMS of compound **2**.

Elemental Composition Report

Page 1

Single Mass Analysis
Tolerance = 5.0 PPM / DBE: min = -5.0, max = 300.0
Element prediction: Off
Number of isotope peaks used for i-FIT = 5

Monoisotopic Mass, Even Electron Ions
2 formula(e) evaluated with 1 results within limits (all results (up to 1000) for each mass)
Elements Used:
C: 15-18 H: 14-20 N: 1-1 O: 3-4 Na: 0-1

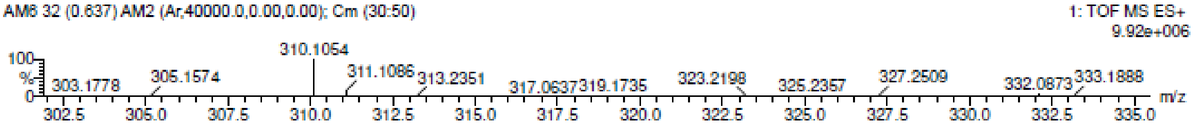

Mass	Calc. Mass	mDa	PPM	DBE	i-FIT	Norm	Conf(%)	Formula
Minimum:				-5.0				
Maximum:		20.0	5.0	300.0				
310.1054	310.1055	-0.1	-0.3	8.5	2681.9	n/a	n/a	C16 H17 N O4 Na

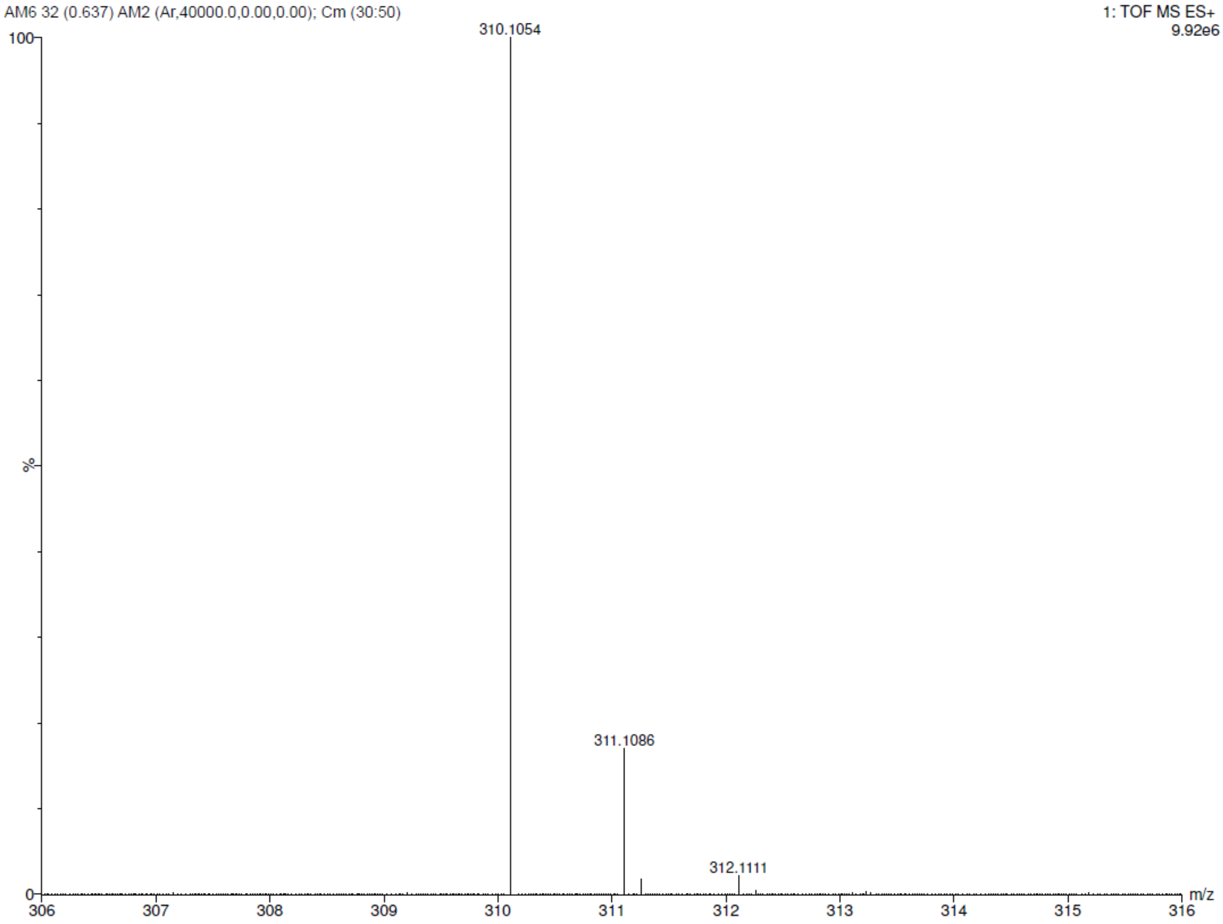

Figure S12. ^{1}H NMR of compound **3**.

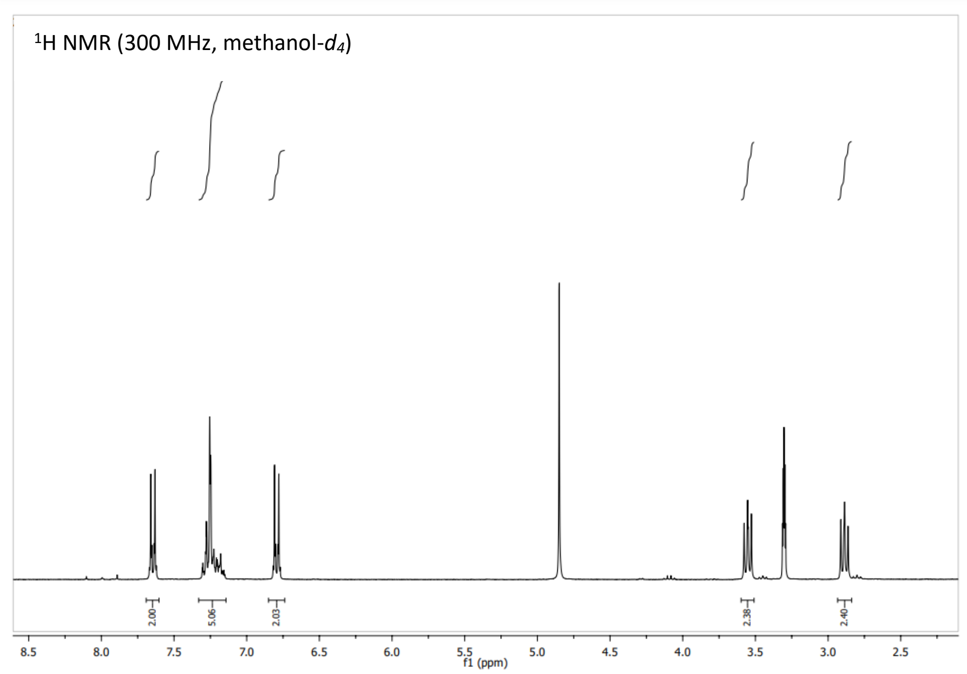

Figure S13. ^{13}C NMR of compound **3**.

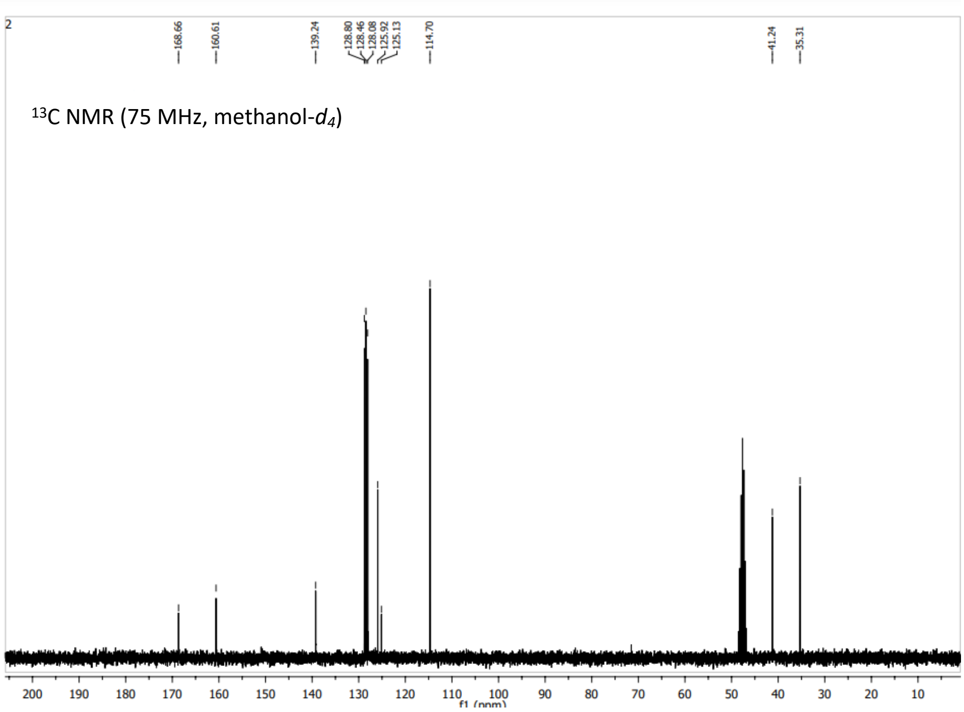

Figure S14. HRMS of compound **3**.

Elemental Composition Report

Single Mass Analysis
Tolerance = 5.0 PPM / DBE: min = -5.0, max = 300.0
Element prediction: Off
Number of isotope peaks used for i-FIT = 5

Monoisotopic Mass, Even Electron Ions
3 formula(e) evaluated with 1 results within limits (all results (up to 1000) for each mass)
Elements Used:
C: 15-18 H: 14-20 N: 1-1 O: 2-4 Na: 0-1

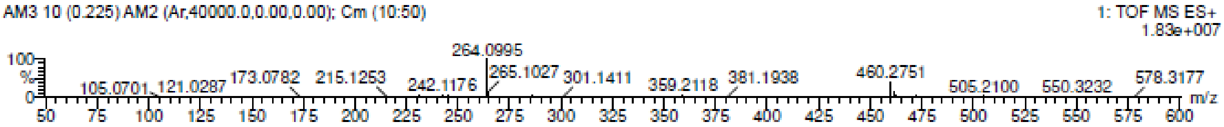

Minimum:				-5.0				
Maximum:		20.0	5.0	300.0				
Mass	Calc. Mass	mDa	PPM	DBE	i-FIT	Norm	Conf(%)	Formula
264.0995	264.1000	-0.5	-1.9	8.5	3132.1	n/a	n/a	C15 H15 N O2 Na

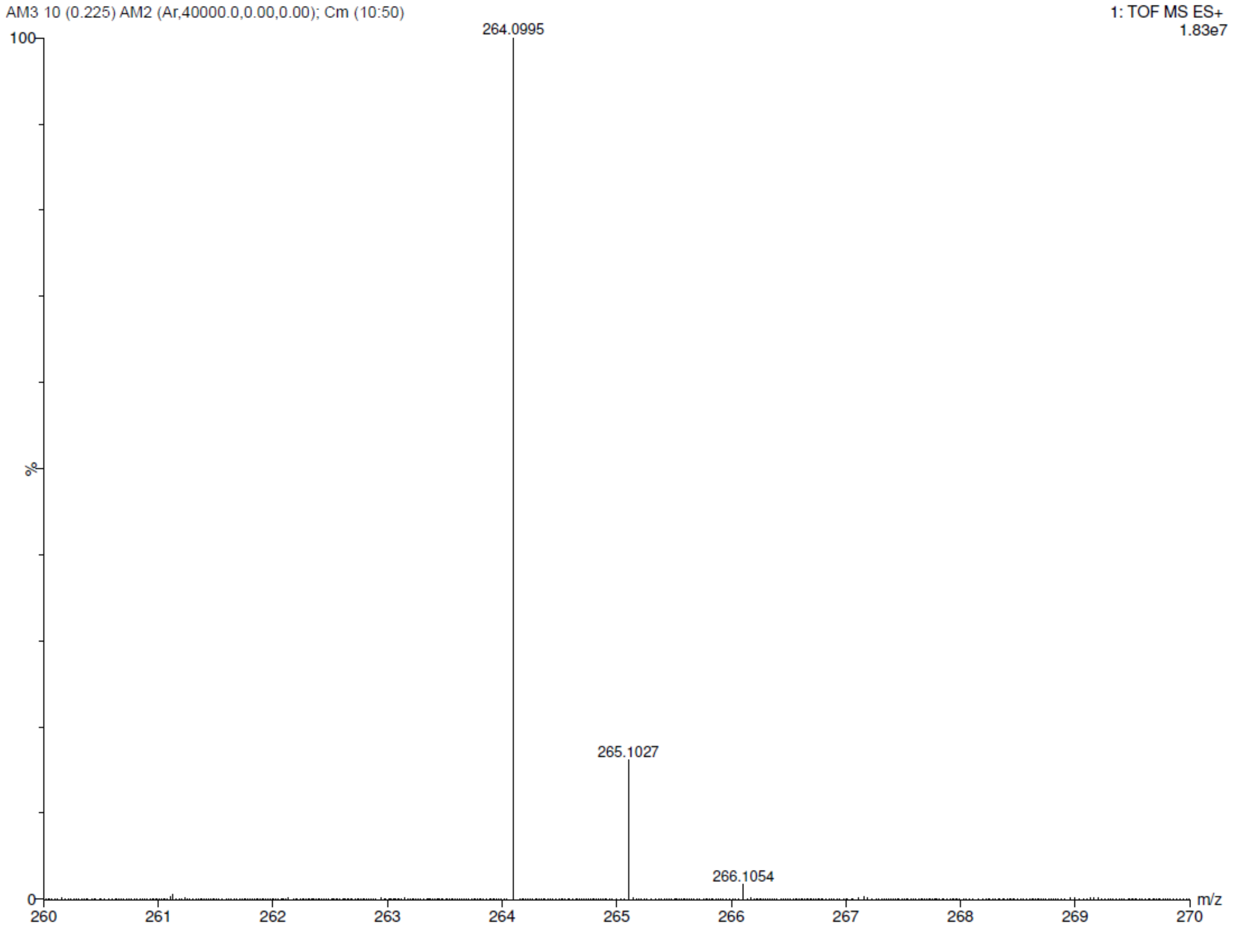

Figure S15. ^{1}H NMR of compound **4**.

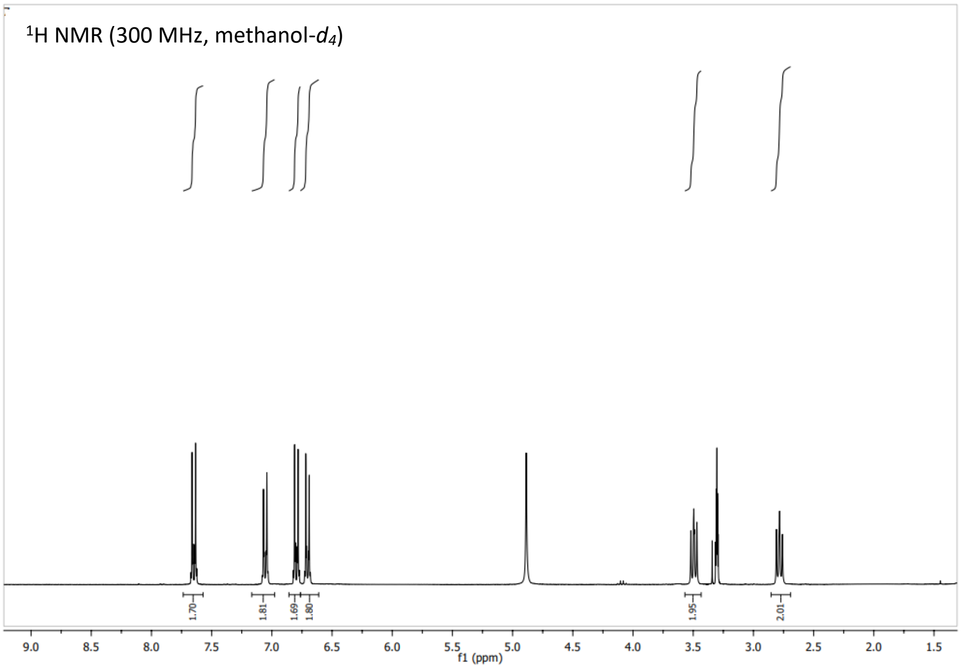

Figure S16. ^{13}C NMR of compound **4**.

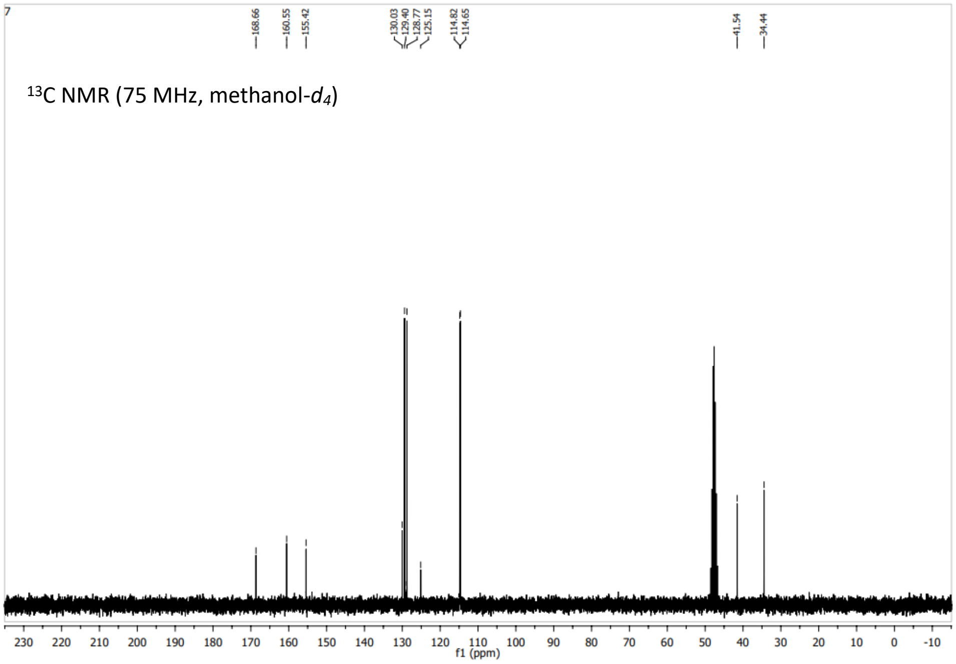

Figure S17. HRMS of compound **4**.

Elemental Composition Report

Page 1

Single Mass Analysis

Tolerance = 5.0 PPM / DBE: min = -5.0, max = 300.0
Element prediction: Off
Number of isotope peaks used for i-FIT = 5

Monoisotopic Mass, Even Electron Ions
2 formula(e) evaluated with 1 results within limits (all results (up to 1000) for each mass)
Elements Used:
C: 15-18 H: 14-20 N: 1-1 O: 3-4 Na: 0-1

AM8 37 (0.741) AM2 (Ar,40000.0,0.00,0.00); Cm (30:50) 1: TOF MS ES+ 9.77e+006

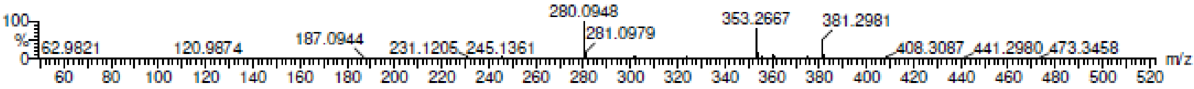

Minimum:				-5.0				
Maximum:		20.0	5.0	300.0				
Mass	Calc. Mass	mDa	PPM	DBE	i-FIT	Norm	Conf(%)	Formula
280.0948	280.0950	-0.2	-0.7	8.5	2997.6	n/a	n/a	C15 H15 N O3 Na

AM8 37 (0.741) AM2 (Ar,40000.0,0.00,0.00); Cm (30:50) 1: TOF MS ES+ 9.77e6

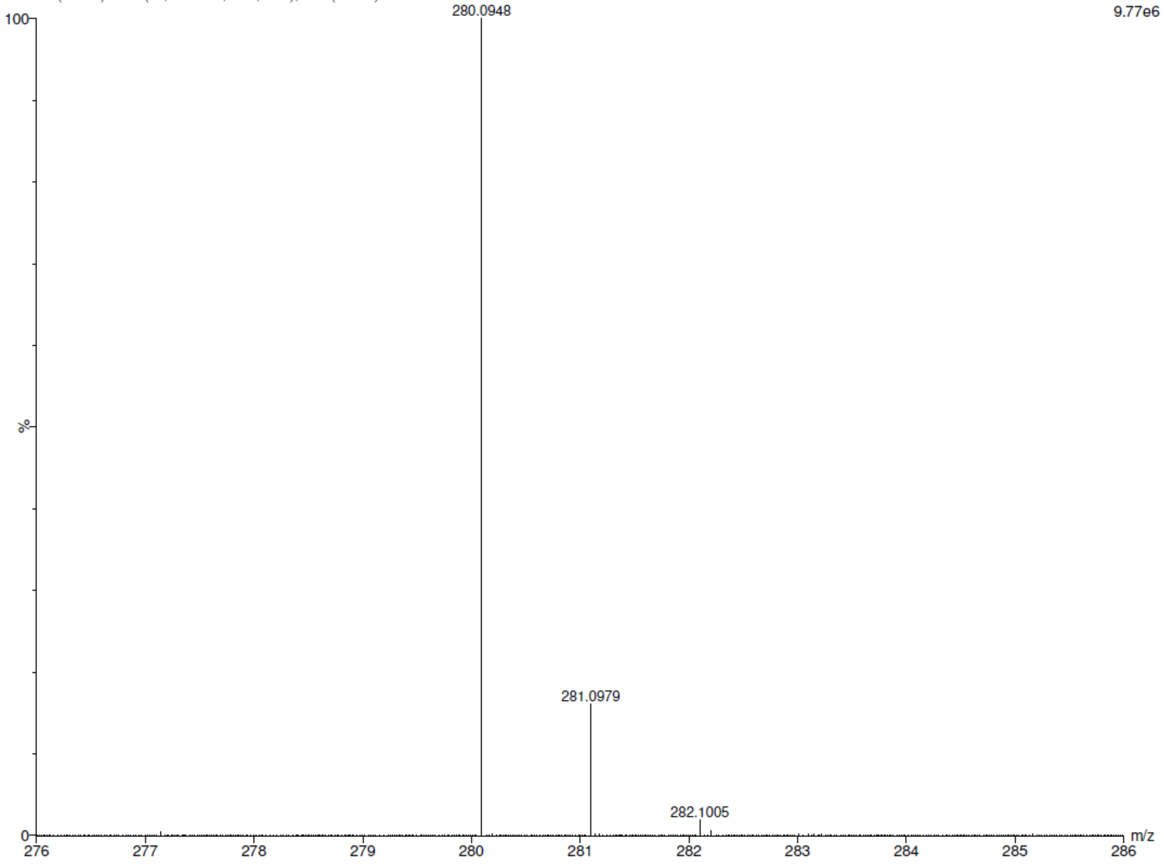

Figure S18. ^{1}H NMR of compound **5**.

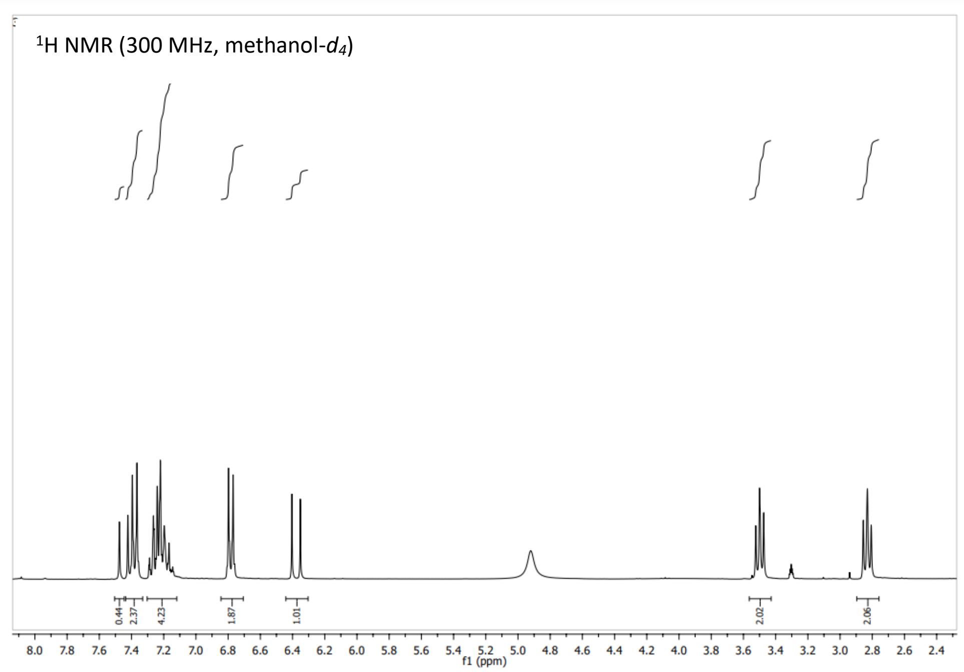

Figure S19. ^{13}C NMR of compound **5**.

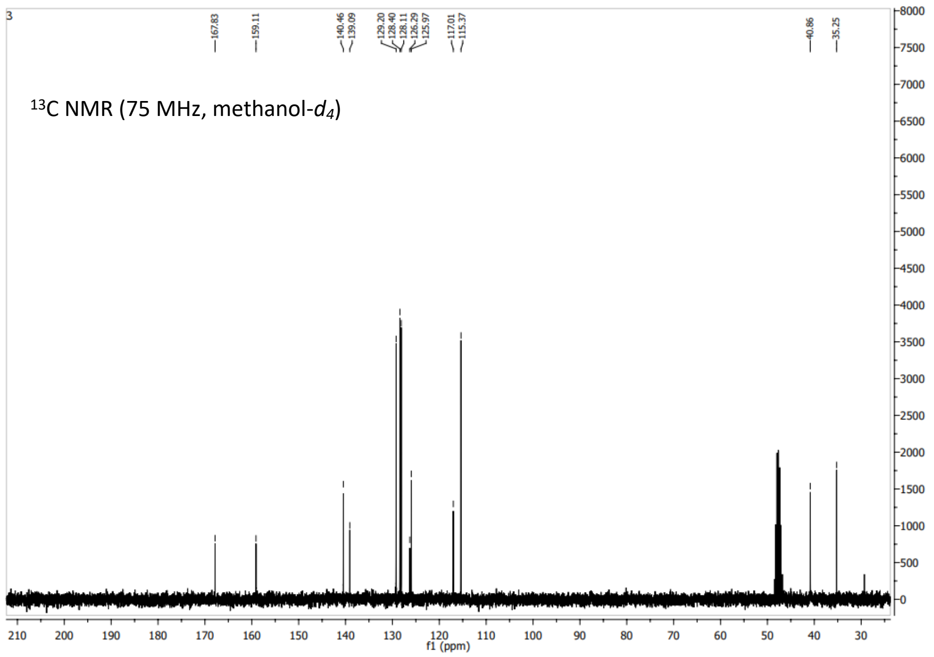

Figure S20. HRMS of compound **5**.

Elemental Composition Report

Page 1

Single Mass Analysis

Tolerance = 5.0 PPM / DBE: min = -5.0, max = 300.0
Element prediction: Off
Number of isotope peaks used for i-FIT = 5

Monoisotopic Mass, Even Electron Ions
2 formula(e) evaluated with 1 results within limits (all results (up to 1000) for each mass)
Elements Used:
C: 15-18 H: 14-20 N: 1-1 O: 2-4 Na: 0-1

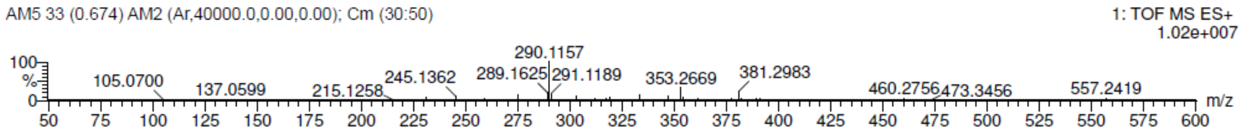

Minimum:				-5.0				
Maximum:		20.0	5.0	300.0				
Mass	Calc. Mass	mDa	PPM	DBE	i-FIT	Norm	Conf(%)	Formula
290.1157	290.1157	0.0	0.0	9.5	2872.3	n/a	n/a	C17 H17 N O2 Na

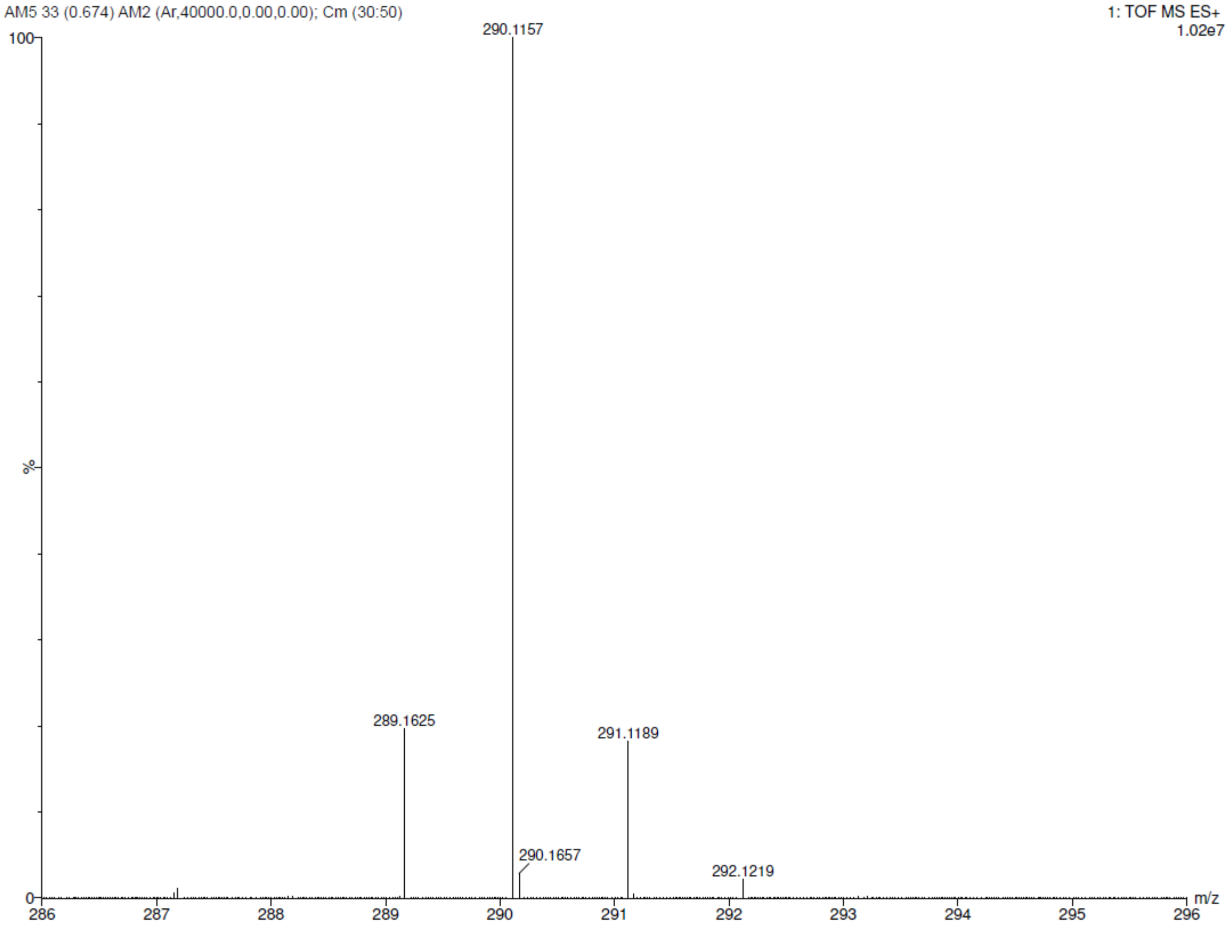

Figure S21. ^{1}H NMR of compound **6**.

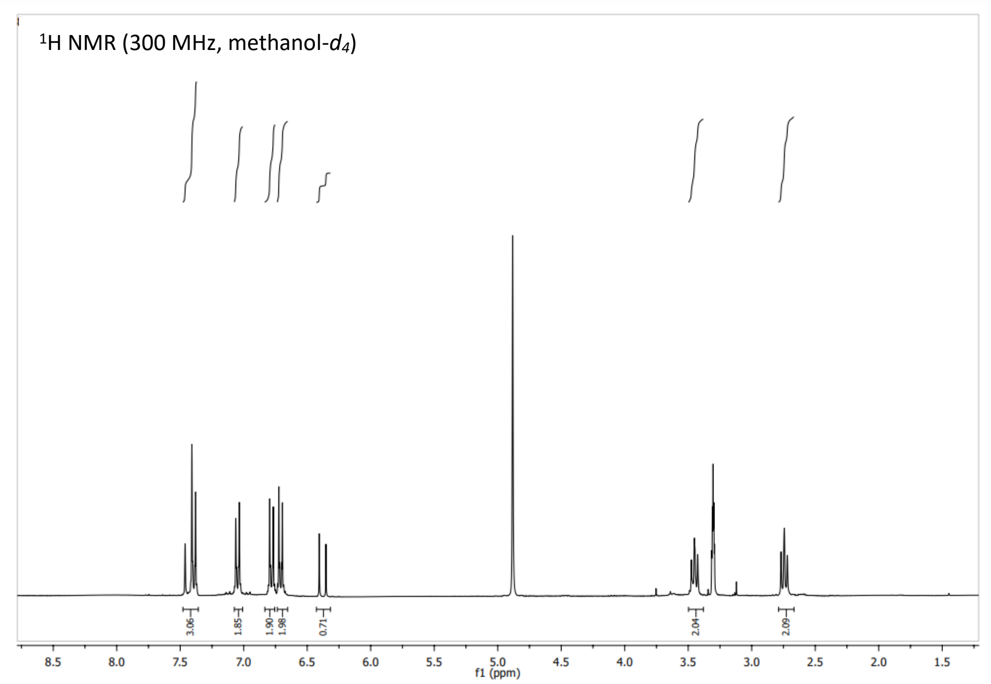

Figure S22. ^{13}C NMR of compound **6**.

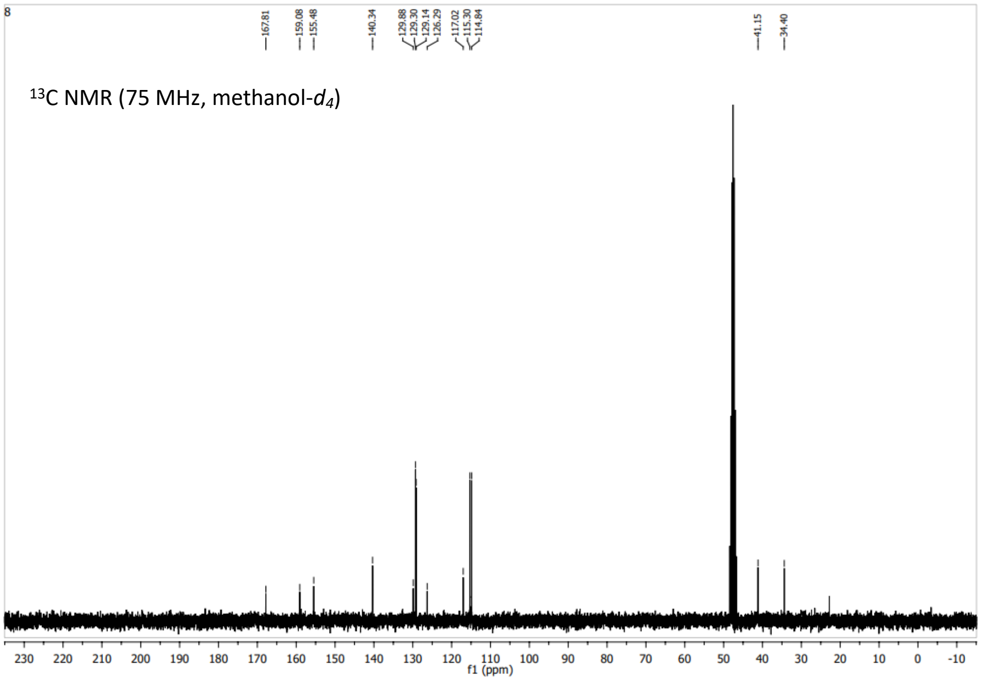

Figure S23. HRMS of compound **6**.

Elemental Composition Report **Page 1**

Single Mass Analysis
Tolerance = 5.0 PPM / DBE: min = -5.0, max = 300.0
Element prediction: Off
Number of isotope peaks used for i-FIT = 5

Monoisotopic Mass, Even Electron Ions
1 formula(e) evaluated with 1 results within limits (all results (up to 1000) for each mass)
Elements Used:
C: 15-18 H: 14-20 N: 1-1 O: 3-4 Na: 0-1
AM10 13 (0.276) AM2 (Ar,40000.0,0.00,0.00); Cm (10:50) 1: TOF MS ES+ 1.87e+007

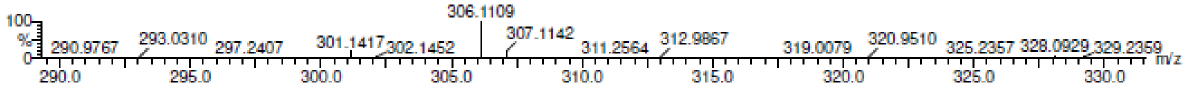

Minimum:				-5.0				
Maximum:		20.0	5.0	300.0				
Mass	Calc. Mass	mDa	PPM	DBE	i-FIT	Norm	Conf(%)	Formula
306.1109	306.1106	0.3	1.0	9.5	3013.6	n/a	n/a	C17 H17 N O3 Na

AM10 13 (0.276) AM2 (Ar,40000.0,0.00,0.00); Cm (10:50) 1: TOF MS ES+ 1.87e7

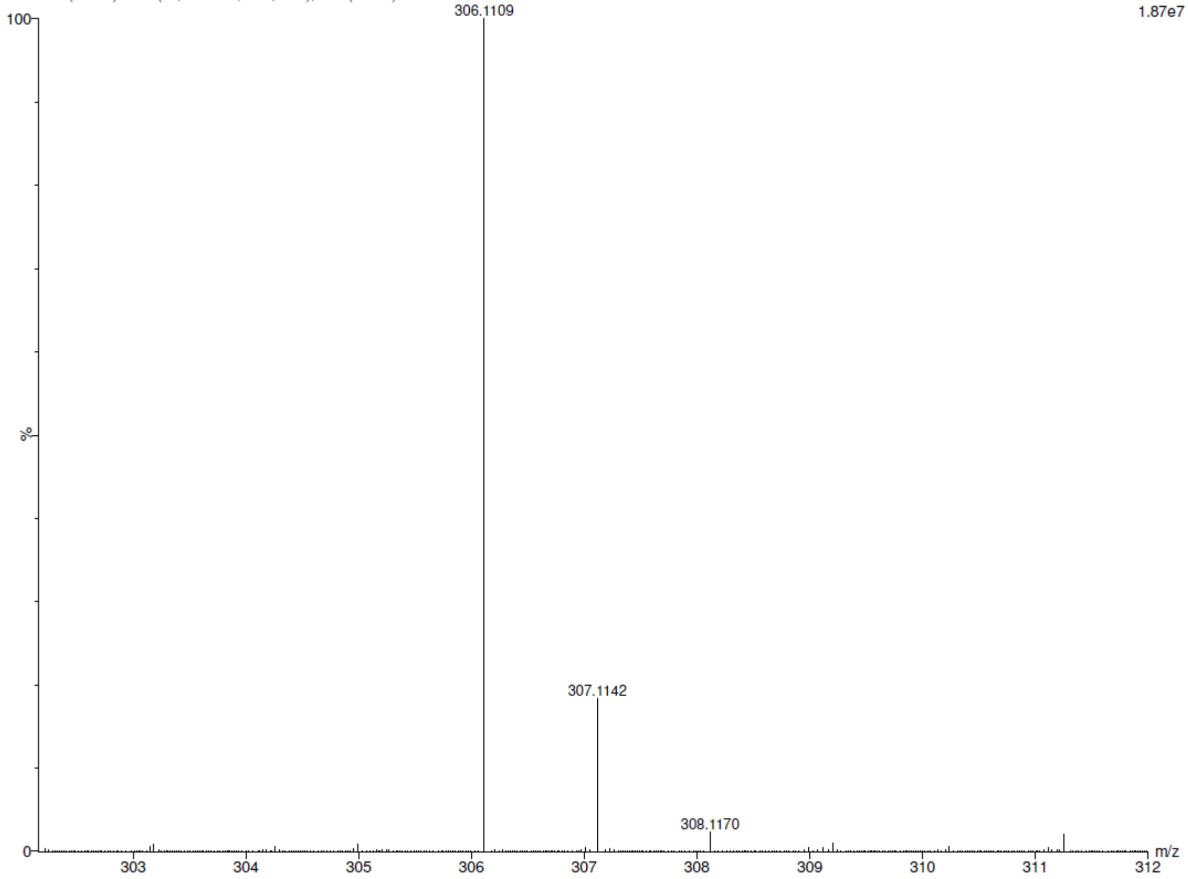

Figure S24. ^{1}H NMR of compound **7**.

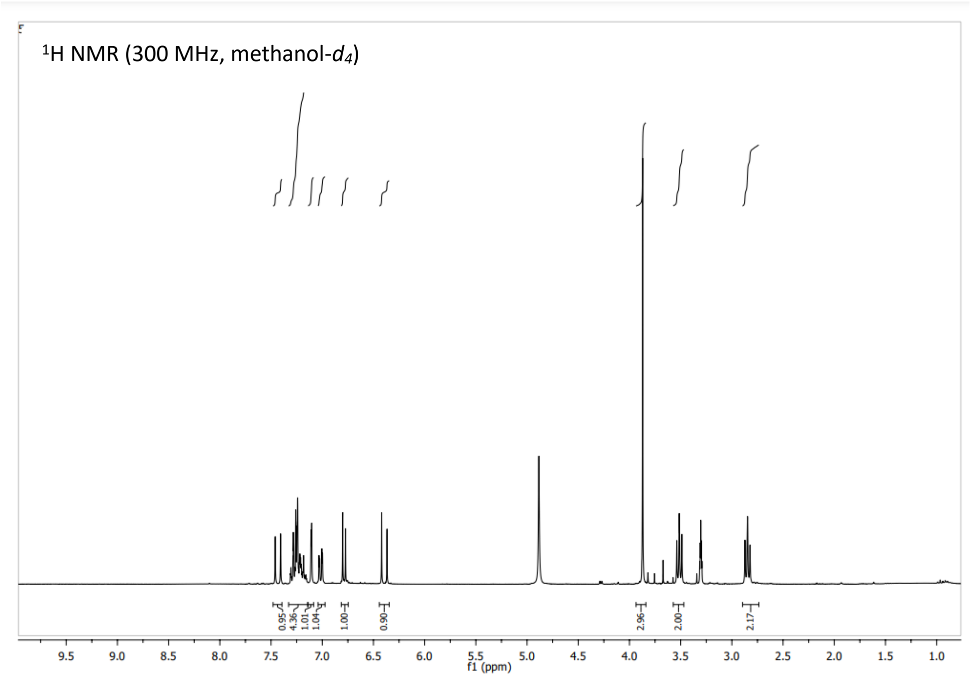

Figure S25. ^{13}C NMR of compound **7**.

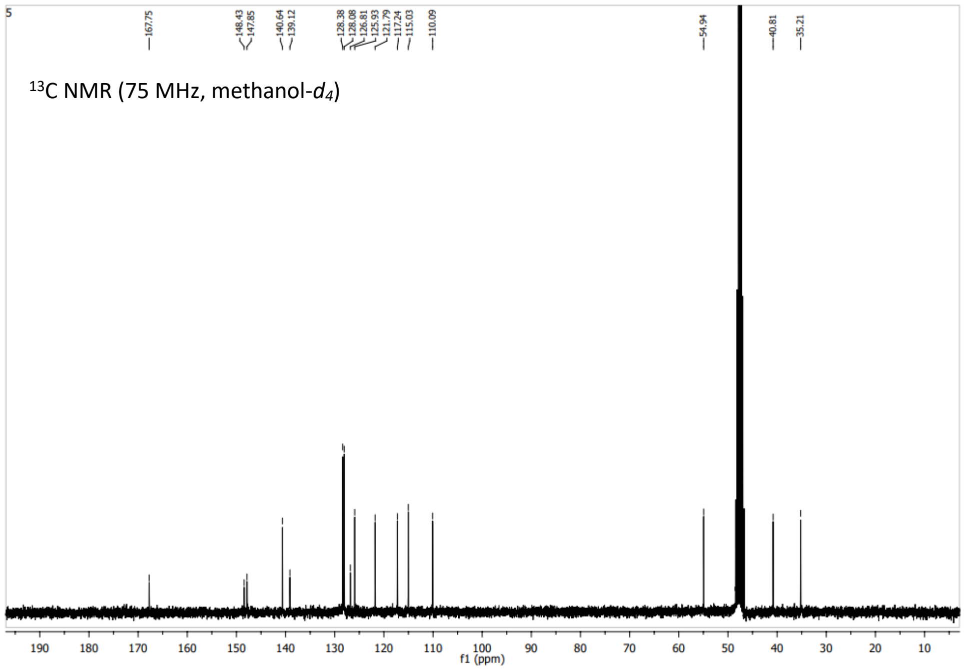

Figure S26. HRMS of compound **7**.

Elemental Composition Report

Page 1

Single Mass Analysis

Tolerance = 5.0 PPM / DBE: min = -5.0, max = 300.0
Element prediction: Off
Number of isotope peaks used for i-FIT = 5

Monoisotopic Mass, Even Electron Ions
3 formula(e) evaluated with 1 results within limits (all results (up to 1000) for each mass)
Elements Used:
C: 15-18 H: 14-20 N: 1-1 O: 2-4 Na: 0-1

AM2 38 (0.758) AM2 (Ar,40000.0,0.00,0.00); Cm (30:50)   1: TOF MS ES+ 1.25e+007

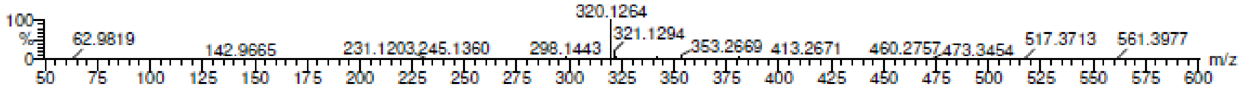

Minimum:				-5.0				
Maximum:		20.0	5.0	300.0				
Mass	Calc. Mass	mDa	PPM	DBE	i-FIT	Norm	Conf(%)	Formula
320.1264	320.1263	0.1	0.3	9.5	2659.9	n/a	n/a	C18 H19 N O3 Na

AM2 38 (0.758) AM2 (Ar,40000.0,0.00,0.00); Cm (30:50)   1: TOF MS ES+ 1.25e7

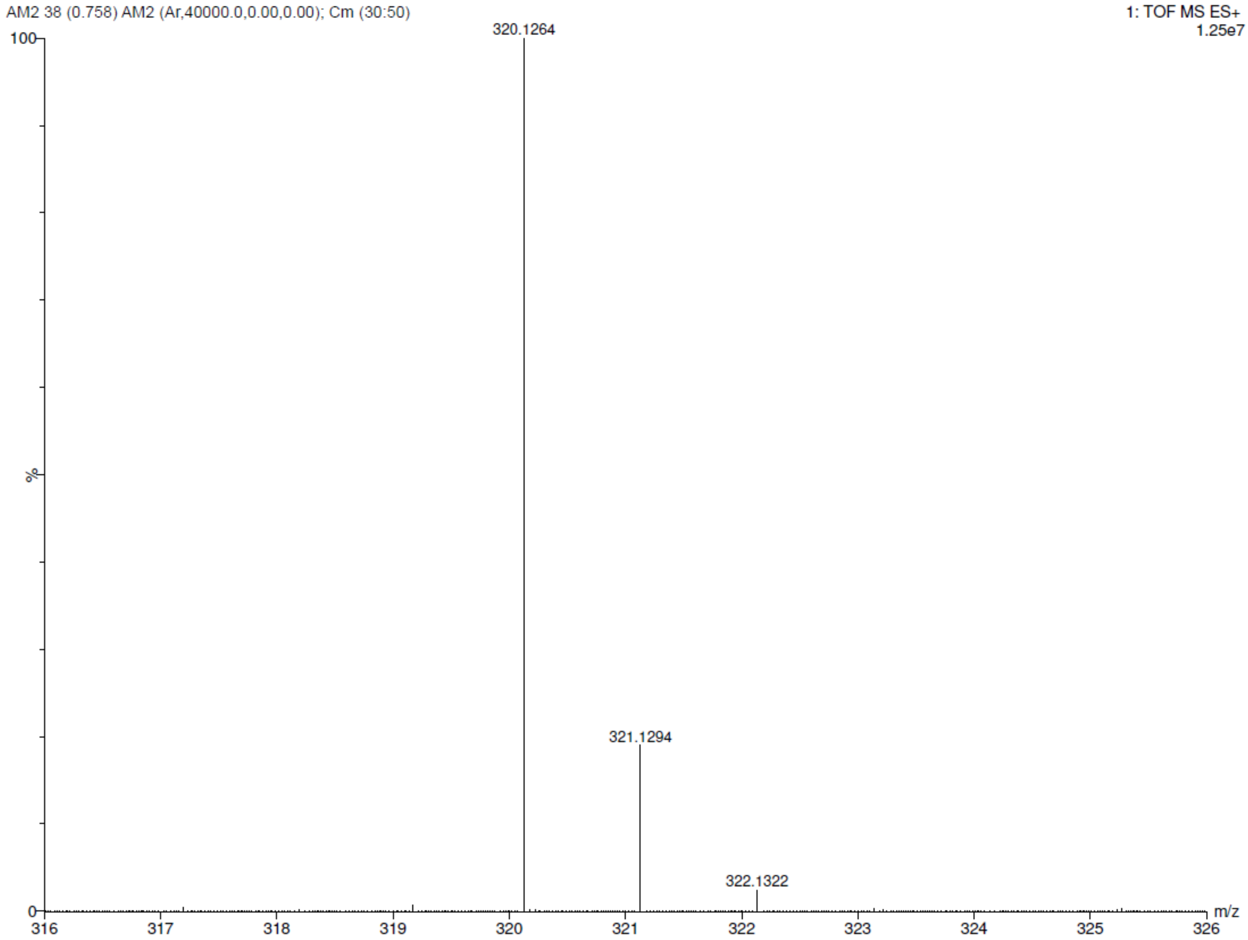

Figure S27. ^{1}H NMR of compound **8**.

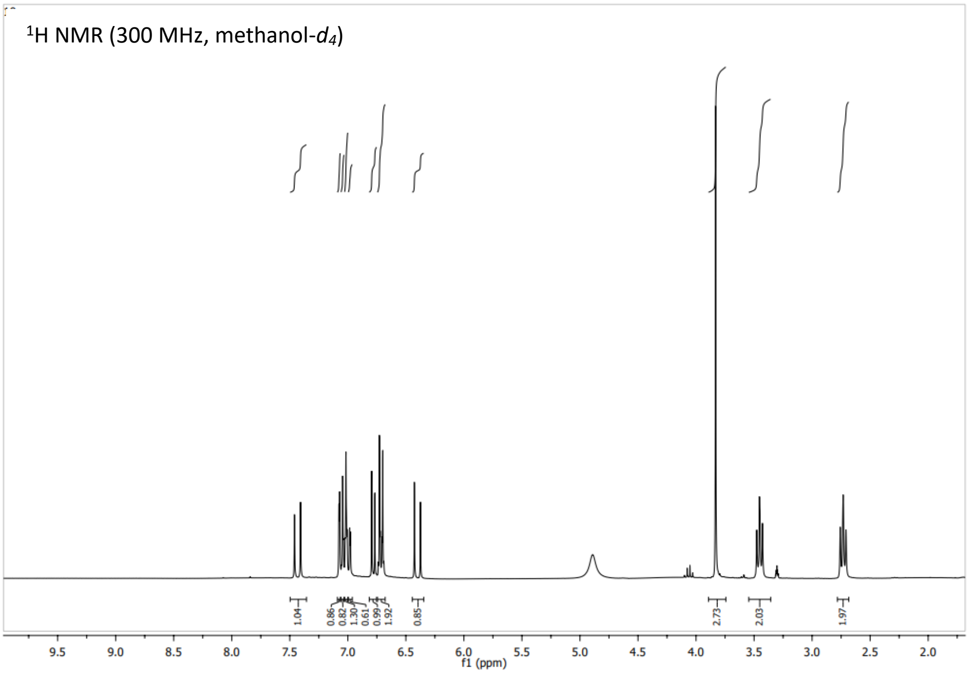

Figure S28. ^{13}C NMR of compound **8**.

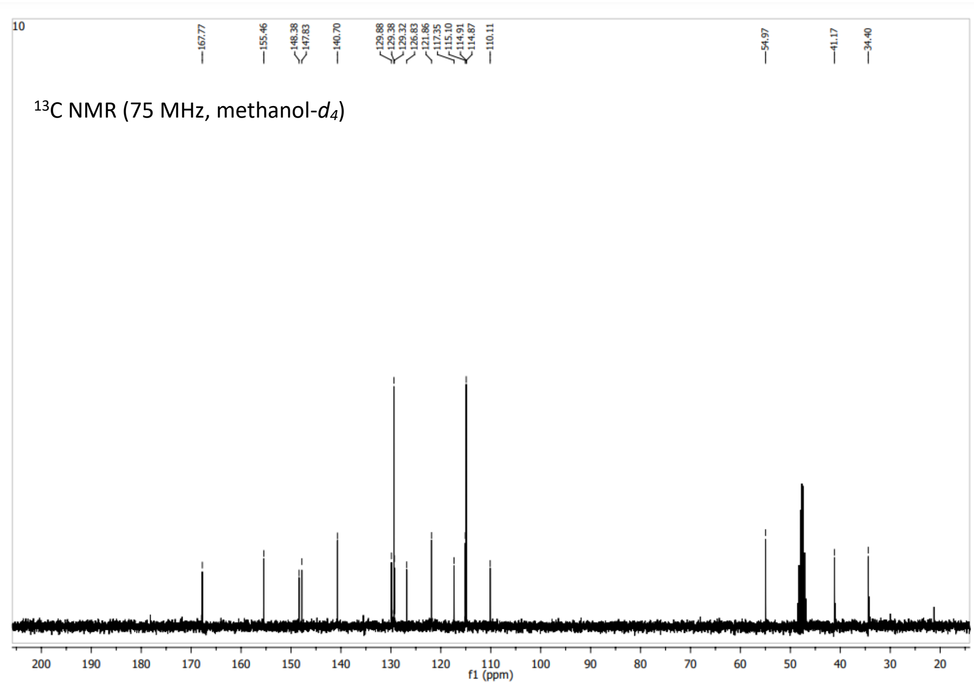

Figure S29. HRMS of compound **8**.

Elemental Composition Report

Page 1

Single Mass Analysis

Tolerance = 5.0 PPM / DBE: min = -5.0, max = 300.0
Element prediction: Off
Number of isotope peaks used for i-FIT = 5

Monoisotopic Mass, Even Electron Ions
2 formula(e) evaluated with 1 results within limits (all results (up to 1000) for each mass)
Elements Used:
C: 15-18 H: 14-20 N: 1-1 O: 3-4 Na: 0-1

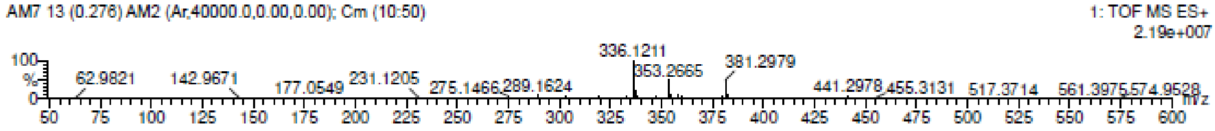

Minimum:				-5.0				
Maximum:		20.0	5.0	300.0				
Mass	Calc. Mass	mDa	PPM	DBE	i-FIT	Norm	Conf(%)	Formula
336.1211	336.1212	-0.1	-0.3	9.5	2847.8	n/a	n/a	C18 H19 N O4 Na

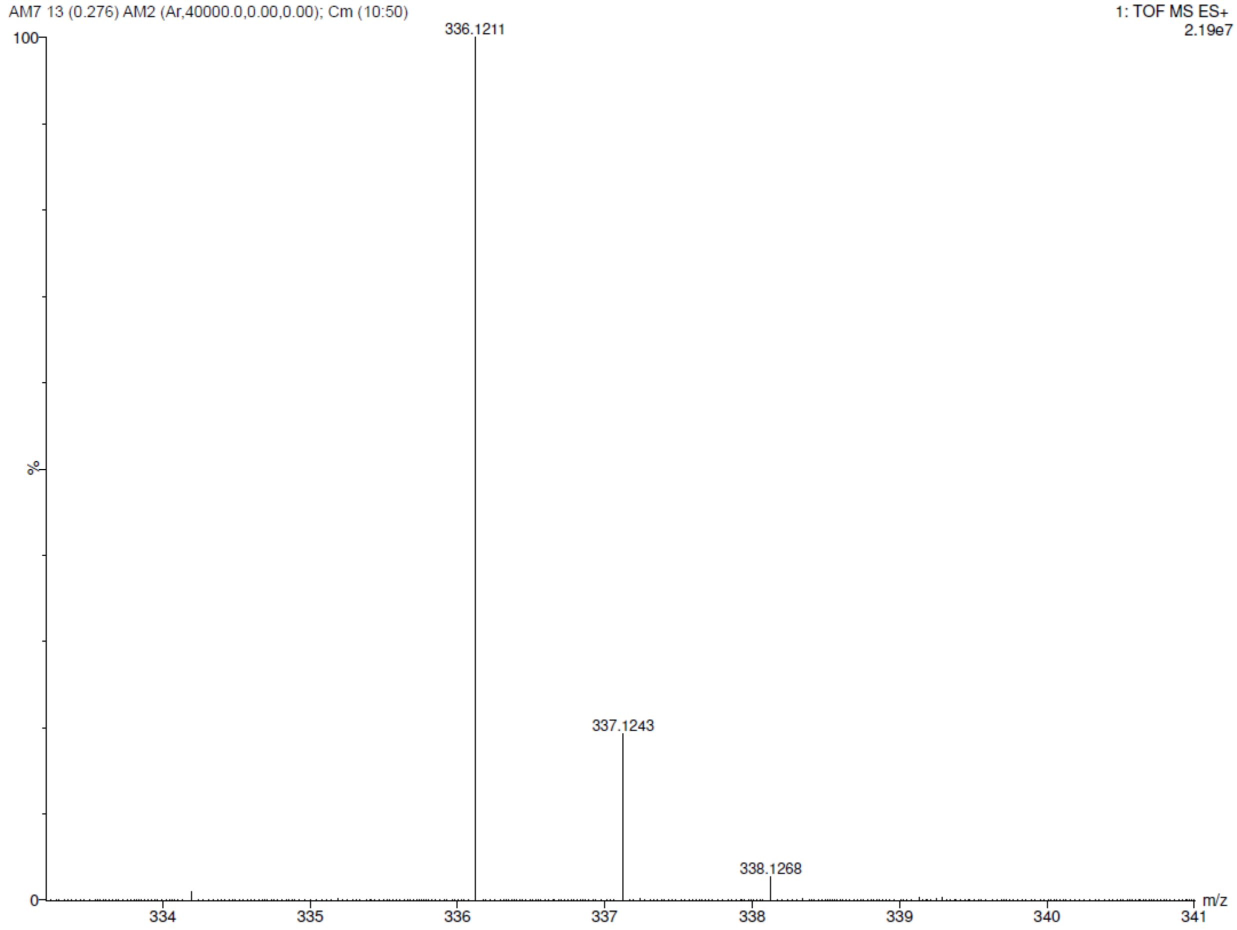

Figure S30. ^{1}H NMR of compound **9**.

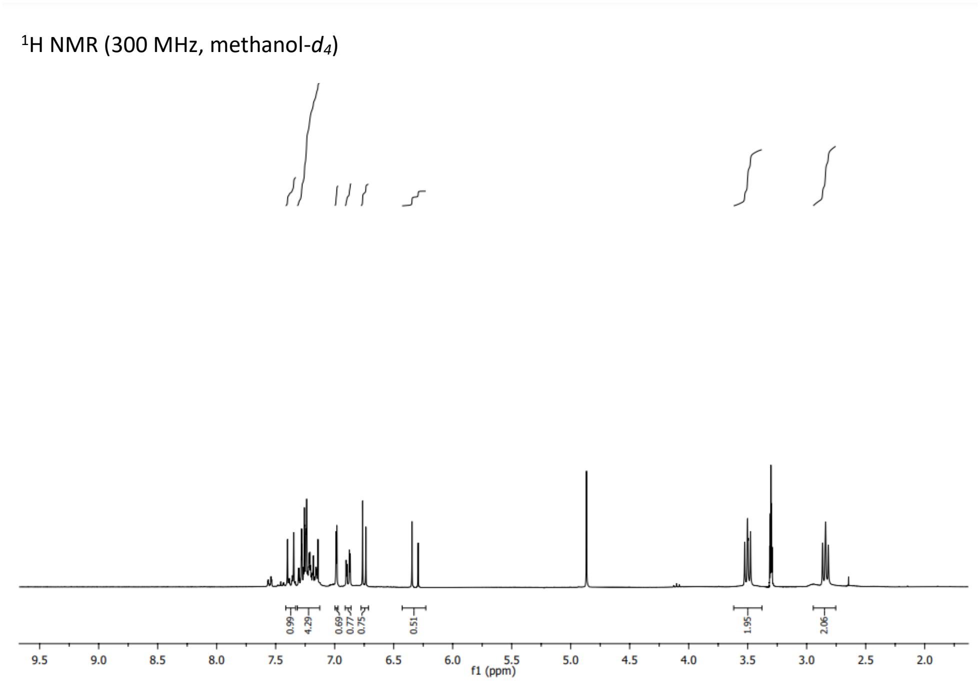

Figure S31. ^{13}C NMR of compound **9**.

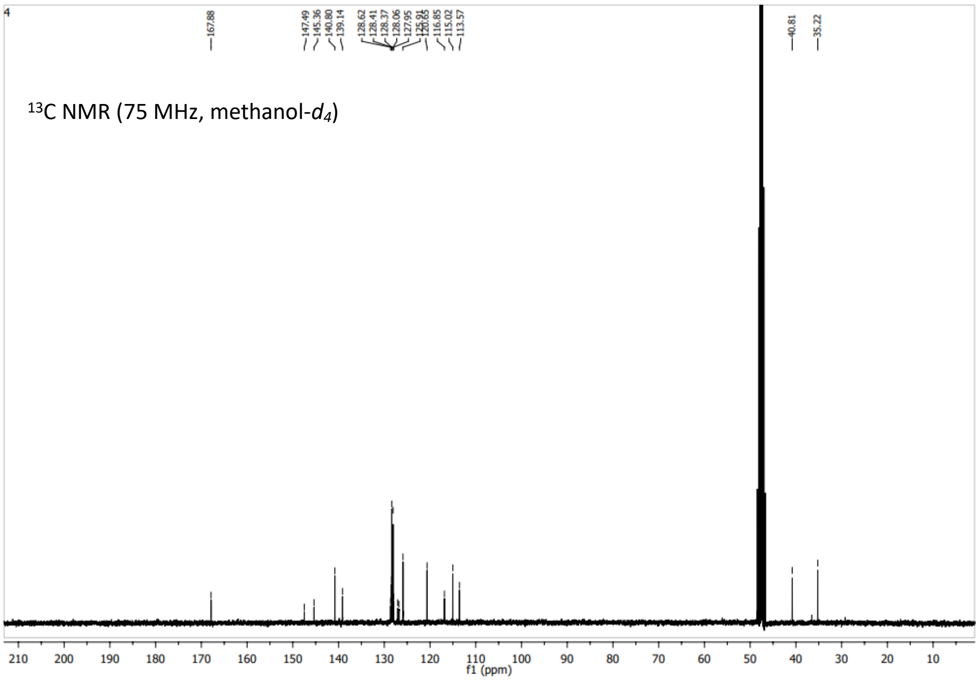

Figure S32. HRMS of compound **9**.

Elemental Composition Report

Page 1

Single Mass Analysis

Tolerance = 5.0 PPM / DBE: min = -5.0, max = 300.0
Element prediction: Off
Number of isotope peaks used for i-FIT = 5

Monoisotopic Mass, Even Electron Ions
1 formula(e) evaluated with 1 results within limits (all results (up to 1000) for each mass)
Elements Used:
C: 15-18 H: 14-20 N: 1-1 O: 2-4 Na: 0-1

AM4 31 (0.620) AM2 (Ar,40000.0,0.00,0.00); Cm (30:50) 1: TOF MS ES+ 8.16e+006

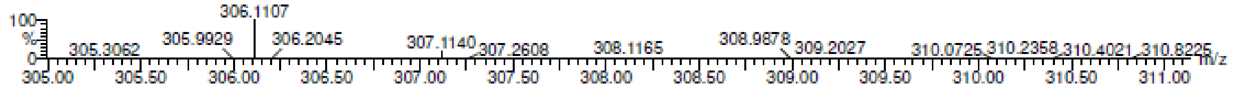

Minimum:				-5.0				
Maximum:		20.0	5.0	300.0				
Mass	Calc. Mass	mDa	PPM	DBE	i-FIT	Norm	Conf(%)	Formula
306.1107	306.1106	0.1	0.3	9.5	2862.3	n/a	n/a	C17 H17 N O3 Na

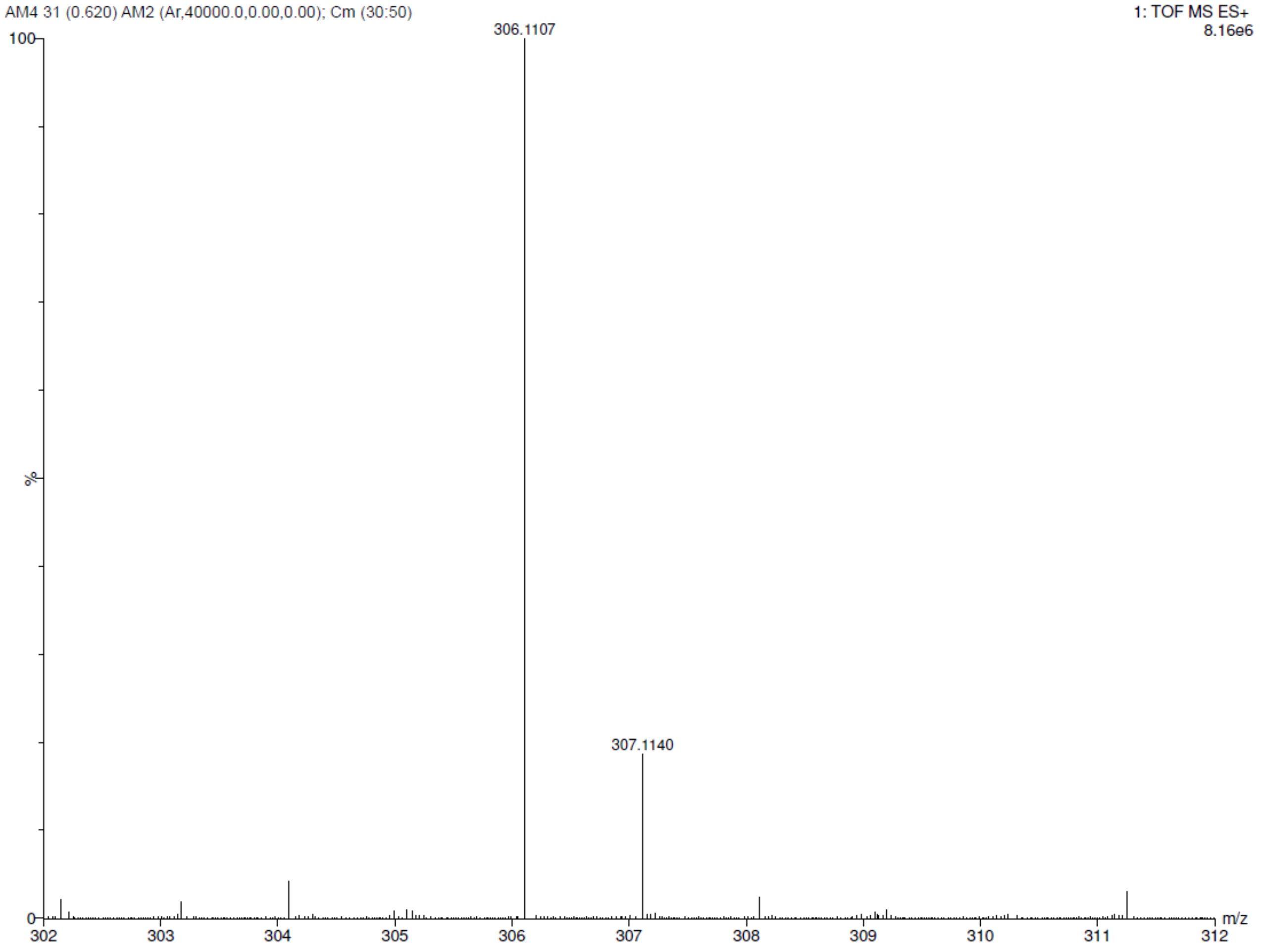

Figure S33. ^{1}H NMR of compound **10**.

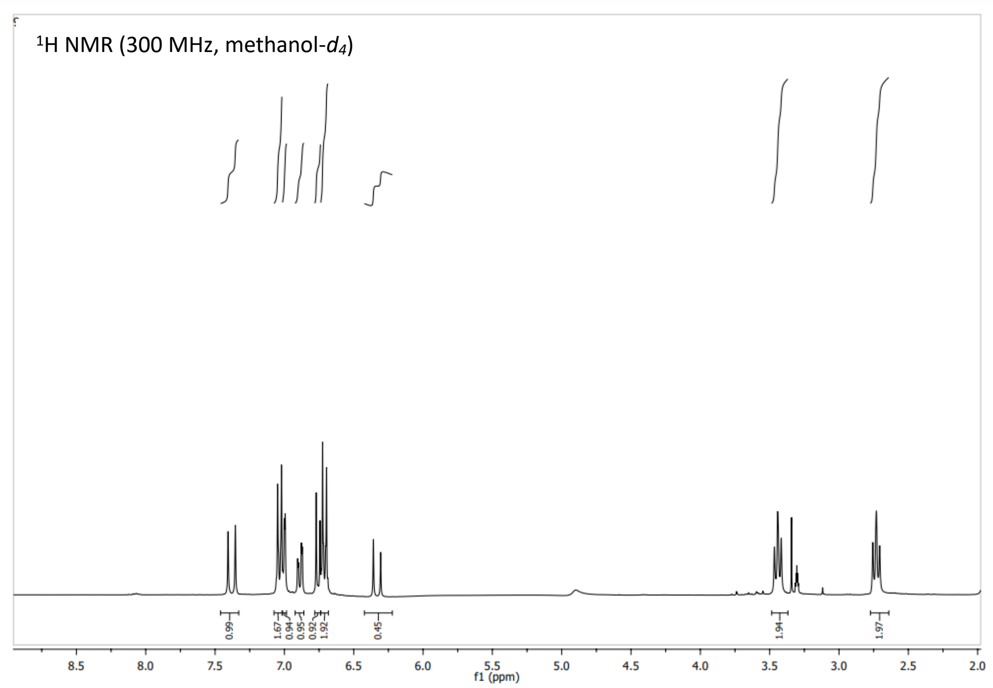

Figure S34. ^{13}C NMR of compound **10**.

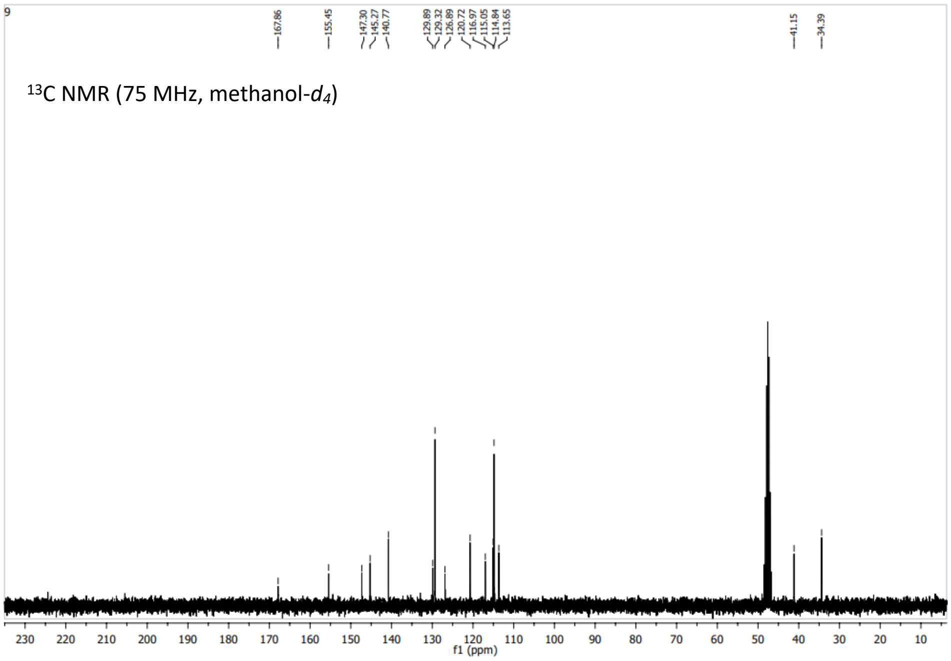

Figure S35. HRMS of compound **10**.

Elemental Composition Report

Single Mass Analysis

Tolerance = 5.0 PPM / DBE: min = -5.0, max = 300.0
Element prediction: Off
Number of isotope peaks used for i-FIT = 5

Monoisotopic Mass, Even Electron Ions
1 formula(e) evaluated with 1 results within limits (all results (up to 1000) for each mass)
Elements Used:
C: 15-18 H: 14-20 N: 1-1 O: 3-4 Na: 0-1

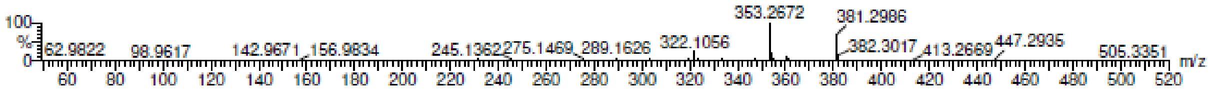

Minimum:				-5.0				
Maximum:		20.0	5.0	300.0				
Mass	Calc. Mass	mDa	PPM	DBE	i-FIT	Norm	Conf(%)	Formula
322.1056	322.1055	0.1	0.3	9.5	2742.5	n/a	n/a	C17 H17 N O4 Na

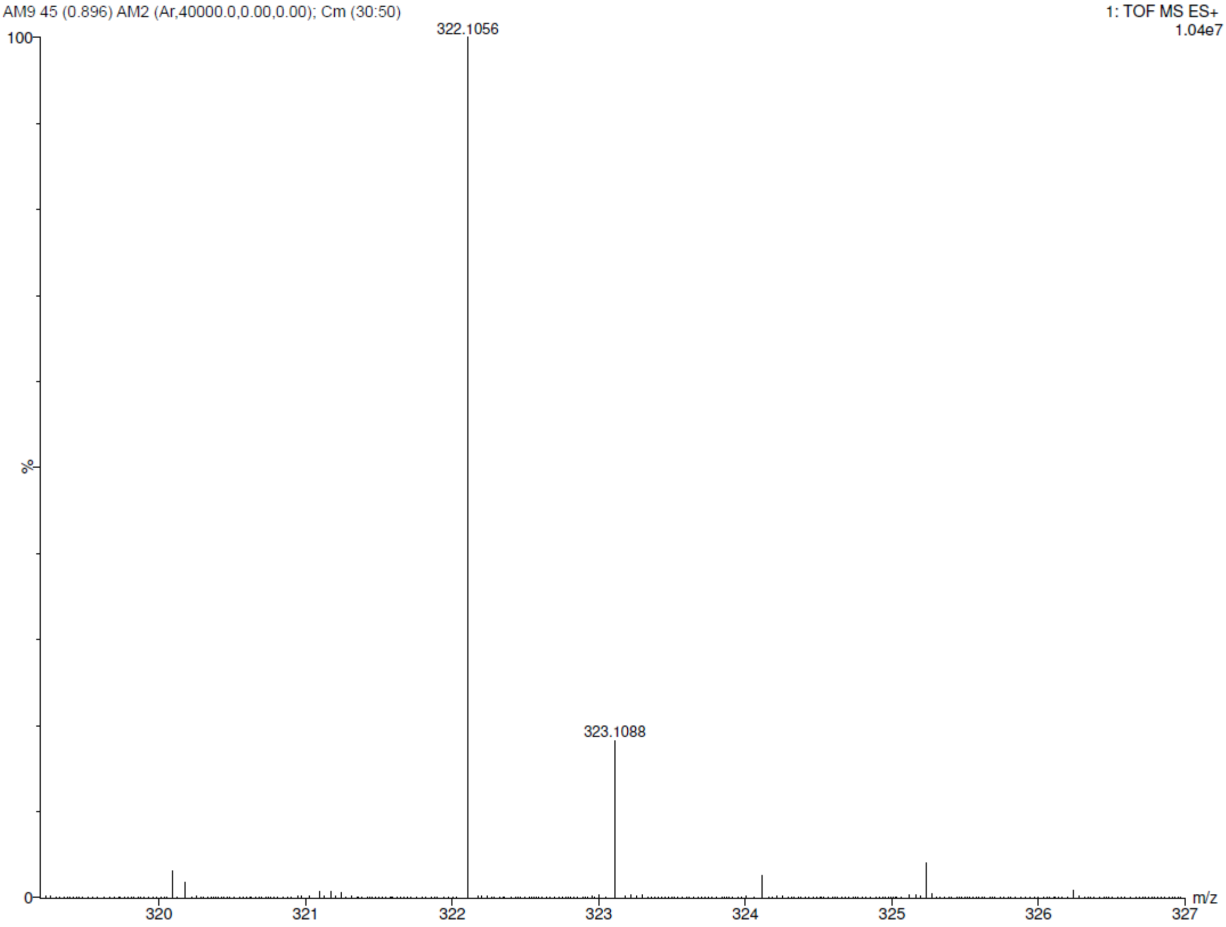

Table S1. Antileishmanial activity towards *L. infantum* and *L. tropica* and calculated LogP of compounds **1-10**.

**Compound**	***L. infantum*** **IC_{50} ± SD (µM)**[a]	**% Inhibition at 100 µM**[b]	***L. tropica*** **IC_{50} ± SD (µM)**[a]	**% Inhibition at 100 µM**[b]	**cLogP**[c]
**1**	>100	5.2	>100	18.3	2.44
**2**	>100	1.8	>100	4.8	2.14
**3**	>100	17.6	>100	36.5	2.69
**4**	>100	0.0	>100	0.0	2.40
**5**	>100	12.1	>100	44.7	3.53
**6**	>100	0.0	>100	0.0	3.24
**7**	16 ± 2	98.2	15 ± 2	98.5	3.27
**8**	>100	3.2	>100	4.4	2.99
**9**	>100	7.1	>100	19.7	3.24
**10**	>100	24.1	>100	0.0	2.96

[a] IC_{50s} are the mean and SD of three experiments in duplicate. [b] % inhibitions are the mean of two experiments in triplicate. [b]Marvin/JChem 20.9 was used to calculate logP values, ChemAxon.
